# Medicinal Herbs Used in Traditional Management of Breast Cancer: Mechanisms of Action

**DOI:** 10.3390/medicines7080047

**Published:** 2020-08-14

**Authors:** Donovan A. McGrowder, Fabian G. Miller, Chukwuemeka R. Nwokocha, Melisa S. Anderson, Cameil Wilson-Clarke, Kurt Vaz, Lennox Anderson-Jackson, Jabari Brown

**Affiliations:** 1 Department of Pathology, Faculty of Medical Sciences, The University of the West Indies, Kingston 7, Jamaica; kurt.vaz@uwimona.edu.jm (K.V.); lennoxwaj@hotmail.com (L.A.-J.); jabarigbrown@gmail.com (J.B.); 2 Department of Physical Education, Faculty of Education, The Mico University College, 1A Marescaux Road, Kingston 5, Jamaica; miller9fabian_gov@yahoo.com; 3 Department of Biotechnology, Faculty of Science and Technology, The University of the West Indies, Kingston 7, Jamaica; 4 Department of Basic Medical Sciences, Faculty of Medical Sciences, The University of the West Indies, Kingston 7, Jamaica; chukwuemeka.nwokocha@uwimona.edu.jm (C.R.N.); cameil.wilsonclarke@uwimona.edu.jm (C.W.-C.); 5 School of Allied Health and Wellness, College of Health Sciences, University of Technology, Kingston 7, Jamaica; melisa_anderson4life@yahoo.com

**Keywords:** breast cancer, herbs, mechanism, action, anti-cancer, chemotherapy, drugs

## Abstract

**Background**: Breast cancer is one of the principal causes of death among women and there is a pressing need to develop novel and effective anti-cancer agents. Natural plant products have shown promising results as anti-cancer agents. Their effectiveness is reported as decreased toxicity in usage, along with safety and less recurrent resistances compared with hormonal targeting anti-cancer agents. **Methods**: A literature search was conducted for all English-language literature published prior to June 2020. The search was conducted using electronic databases, including PubMed, Embase, Web of Science, and Cochrane Library. The search strategy included keywords such as breast cancer, herbs, anti-cancer biologically active components, clinical research, chemotherapy drugs amongst others. **Results**: The literature provides documented evidence of the chemo-preventative and chemotherapeutic properties of Ginseng, garlic (Allium sativum), Black cohosh (Actaea racemose), Tumeric (Curcuma longa), Camellia sinenis (green tea), Echinacea, Arctium (burdock), Flaxseed (Linum usitatissimum) and Black Cumin (Nigella sativa). **Conclusions**: The nine herbs displayed anti-cancer properties and their outcomes and mechanisms of action include inhibition of cell proliferation, angiogenesis and apoptosis as well as modulation of key intracellular pathways. However, more clinical trials and cohort human studies should be conducted to provide key evidence of their medical benefits.

## 1. Introduction

Breast cancer is a recognized adenocarcinoma, the most common cancer in women and the second leading cause of death in women after lung cancer. Mortality impacts are somewhat ambiguous, and often linked to socioeconomic and lifestyle status [1,2]. It is a very heterogeneous disease with variation in histological grade, proliferative index (Ki67), immunohistochemistry and clinical presentation [3]. The Ki67 and the Bloom–Richardson scoring system are useful in the prediction of the levels of tumor aggression, even though the same histological subtypes have different presentations in different age groups, marked by different fertility related factors, which could also affect the proliferation and management of the disease. These include fertility damage, sexual dysfunction and menopausal symptoms [4].

Therapy and outcomes for breast cancer are often dependent on the subtypes (hormone receptor positive, Her 2 amplified and triple-negative subtypes), and involve hormonal, radiotherapy, molecular and chemotherapy interventions. Surgery has become increasingly advised, especially for metastatic situations [5,6,7]. The stage of breast cancer is also predictive of survival and depends on the size of the primary breast tumor, axillary lymph node involvement as well as distant metastasis [8]. Such heterogeneity in presentation and treatment outcomes has led to a multimodal and multidisciplinary approach that involves the use of natural plant product, so as to enhance survivability. These multimodal approaches are still dependent on early detection and disease diagnosis [9].

Natural plant products have shown promising results as anti-tumor and anti-cancer agents. Their effectiveness is also reported as decreased toxicity in usage, and less recurrent resistances to hormonal targeting anti-cancer agents (multidrug resistances as seen with several anti-cancer agents) [10,11]. Such uses are due to their antioxidant and anti-inflammatory properties, coupled with their immunomodulatory properties, and abilities to induce anti-proliferative and anti-apoptotic effects on these cancer cells. This is done in a manner to present a chemo-preventative property, which can be prophylactic and therapeutic, and are safe for long term usage [12].

Constituents of natural plant products such as flavonoids, alkaloids, terpenoids, coumarins are known for their antioxidant and anti-inflammatory properties (glabridin, curcumin, arctigenin and ajoene) and lymphocytes activation (quinic acid, β-carotene, epigallocatechin-3-gallate, and ginsan), which are strong immunomodulatory properties needed to suppress, or fight against cancer cells [13]. Bioactive compounds like phytoestrogens (non-steroidal phenolic compounds with structural similarities comparable with steroids like oestrogen), and isoflavinoids can act as endocrine disrupters for hormonal disorders, which are often the basis for the presentation of these cancer outcomes. These plant flavonoids are reported to possess estrogenic and or anti-estrogenic properties, which are also chemo-preventative properties [14,15]. They are able to inhibit oestrogen receptor dependent (cell growth and proliferations) and independent (generation of free radicals and genotoxic agents) associations and possess the ability to induce oxidative stress and cancer induction through oestrogen receptor signaling [16].

There are studies that have provided evidence of the efficacy of natural products such as herbs in the development of anti-cancer drugs. This review is centered on the biochemical properties and pharmacokinetics of Ginseng, garlic (*Allium sativum*), Black cohosh (*Actaea racemose*), Tumeric (*Curcuma longa*), *Camellia sinenis* (green tea), Echinacea, *Arctium* (burdock), Flaxseed (*Linum usitatissimum*) and Black Cumin (*Nigella sativa*) which possess chemo-preventative and chemotherapeutic properties. These well-known herbs have been selected as they are commonly used in traditional medicine as adjuvants in breast cancer therapy and there is documentation of their mechanisms of action.

This review also examined the mechanism(s) of action and the modulatory role of these herbs of key intracellular signaling pathways involved in the development and progression of breast cancer. In addition, current limitations of these herbs, challenges and future directions for experimental in vitro and in vivo techniques, animal models and clinical research are critically appraised.

## 2. Ginseng

### 2.1. Different Types of Ginseng and its Preparation

Ginseng is a well described perennial herb that belongs to the Araliaceae family and *Panax* genus [17]. The species of plants that comprise ginseng are *Panax japonicus* (Asian ginseng), *Panax quinquefolius* L. (American ginseng) and *Panax ginseng* [18]. *Panax ginseng* C.A. Meyer (Korean or Asian ginseng) is the most frequently used species and is cultivated in Korea and China, while *Panax quinquefolius* was grown originally in Canada and the United States of America [19]. In the last two decades, ginseng has become recognized as one of the most frequently used alternative and complementary herbal medicines in the West, and significant research has been conducted on *Panax ginseng* C.A. Meyer [20].

Ginseng possesses a sweet taste and can be categorized based on the manner in which it is processed. Ginseng that is fresh and during processing is steamed once is known as Red ginseng (Ginseng Radix Rubra), repeatedly steamed nine times is called Black ginseng (Ginseng Radix Nigra) and dried referred as White ginseng (Ginseng Radix Alba). The fine roots of the Ginseng plant such as *Panax ginseng* Meyer consist of ginsenosides, the main active chemical compounds, and over 40 other constituents have been recognized and isolated from the *Panax* species [21,22]. Ginsenosides are triterpene steroidal saponins and are regarded as being different based on the location, type and number of their sugar moieties such as glucose, rhamnose, arabinose or xylose.

Ginsenosides of ginseng are categorized based on the chemical structure of their aglycones and there are three main types, namely: (i) protopanaxadiol group, known as diols, which include Rb1, Rb2, Rb3, Rc, Rd, Rg3, Rh2 and Rs1, (ii) protopanaxatriol group or triols, including Re, Rf, Rg1, Rg2, and Rh1 and (iii) oleanane group which is comprised mainly of Ro [23,24].

The *Panax* species are differentiated by the relative quantities of ginsenosides. The most prevalent ginsenosides that comprised more than 90% of the total content of the root of *Panax ginseng* are Rg1, Re, Rd, Rc, Rb3 and Rb1. *Panax ginseng* also has greater levels of Rg1 compared with Rb1 and thus a higher ratio of Rg1/Rb1 compared with American ginseng [25]. The root of the American ginseng consists mainly of Rg1, Re, Rd, Rc, Rb3 and Rb1 that account for greater than 70% of the total content [26]. The pharmacology and mechanism of action of ginsenosides vary due to their different chemical structures and in purified forms, those that are commonly investigated particularly for their anti-cancer activities are Rb1, Rg1, Rg3 and Rh1 and Rh2 [27]. In addition to ginsenosides, ginseng consists of acidic and neutral water-soluble polysaccharides present in 15% of the root [28].

### 2.2. The in Vitro Anti-Tumor Effects of the Bioactve Compounds of Ginseng

The ginsenoside Rh2 is a significant bioactive constituent of red ginseng and the main active anti-cancer saponin in extracts [29]. Lee et al. [30] investigated the inhibitory effect of Rh2 on the growth of breast cancer cells in vitro by using MCF-7, a breast cancer cell line. They reported that Rh2 retarded the proliferation of the MCF-7 human breast cancer cell line in a dose-dependent manner by inducing changes in hypo-methylated genes involved in tumorigenesis with the upregulation of ST3GAL4, C1orf198 and CLINT1 [30]. A similar mechanism by which Rh2 exerts in anti-cancer activity involves the suppression of C3orf67-AS1, a novel noncoding RNA via promotor methylation [31].

Rh2 significantly inhibits the growth of MDA-MB-231 and MCF-7 breast cancer cell lines in a concentration-dependent manner by mediating G(0)/G(1) phase cell cycle arrest. The mechanism of significant inhibition of both cell lines by Rh2 is via p27(Kip1) and p15(Ink4B)-dependent inhibition of activities of kinases of G(1)-S specific Cdks/cyclin complexes, particularly cyclin (cyclin D1/Cdk6 and cyclin D1/Cdk4) [32] (Table 1). Similar results were reported in an earlier study where Rh2 inhibited the proliferation of MCF-7 human breast cancer cell line by increasing the protein expression of p21 and decreasing protein levels of cyclin D, resulting in lower phosphorylation of pRb and down-regulation of cyclin/Cdk complex kinase activity [33]. Furthermore, the mechanism by which Rh2 exerts its anti-cancer activity involves inhibiting the manufacture of inflammatory cytokines such interleukin (IL)-1β and tumor necrosis factor (TNF)-α through obstructing the nuclear factor (NF)-κB signaling and mitogen-activated protein kinase pathways in MCF-7 breast cancer cells [34,35].

The ginsenoside Rg3 is one of the chief active compounds derived from heat processing of ginseng, and one of its significant function is the inhibition of proliferation of cancer cells. In vitro, 20(S)-Rg3 significantly inhibits the proliferation of MDA-MB-231 and MCF-7 breast cancer cells by decreasing the expression of cyclin D1 and cyclin A as well as arresting the cells in the G-1 phase [36] (Table 1). Two recent studies reported that Rg3 inhibits the growth of breast cancer cells by the deregulation of the tumor-related genes NOX4 and KDM5A by modification of the epigenetic methylation levels [47], and through phospho-proteomic analysis, Rg3 regulated a number of central inhibitors of nuclear factor-κB signaling, cell division and protein synthesis [48].

One of the most studied molecular outcomes of the anti-cancer action of Rg3 is apoptosis. Rg3 induced apoptosis in MDA-MB-231 breast cancer cell line via activating of the proteolytic cleavage caspase-3 enzymes and degrading the poly (ADP-ribose) polymerase through the production of reactive oxygen species. In addition, Rg3 increases the pro-apoptotic Bax and decreases the anti-apoptotic Bcl-2 [49]. In another study Rg3 induces apoptosis in MDA-MB-231 breast cancer cell line by blocking NF-κB signaling via the inactivation of Akt and ERK kinases, resulting in decreased cell proliferation and cell cycle progression [50]. Rg3 in inducing apoptosis destabilizes the mutant tumor suppressor protein p53 which can extend the activation of the transcription factor NF-κB [50].

Insulin-like growth factors (IGFs), particularly IGF-1 has a critical role in the growth and development of breast cancer. It is reported that Rg3 inhibits tumor growth and angiogenesis by degrading levels of IGF-1 in an animal model [51]. In a study by Chen et al. 20(S)-Rg3 reduced the expression of the chemokine receptor CXCR4 in MDA-MB-231 breast cancer cells [52]. The CXCR4 receptor expressed by breast cancer cells and its ligand CXCL12, has a critical role in the metastasis. By decreasing the expression of CXCR4, Rg3 decreases metastasis to bones, lungs and lymph nodes [52,53].

### 2.3. Effects of Ginseng in Combination with Anti-Cancer Drugs

There are only a few studies that have reported the effects of the co-administration of a chemotherapy agent and Rg3 in breast cancer models [54,55]. The co-administration of 20(s)-ginsenoside Rg3 orally and paclitaxel enhanced the relative bioavailability of paclitaxel and reduce the growth rate of the breast tumor in nude mice with MCF-7 xenograft [54] (Table 2). In another study mice administered with capecitabine a novel fluoropyrimidine carbamate, fluorouracil (5-FU), and Rg3 exhibited less toxicity caused by capecitabine, decreased susceptibility to drug resistance and enhanced better survival in mice with breast cancer [55]. Rg3 enhanced the antiangiogenic effects of capecitabine by decreasing microvasculature density and the expression of vascular endothelial growth factor (VEGF) in a mice model [55].

### 2.4. Clinical Studies with Ginseng

Studies have found that cancer patients consume ginseng for a number of reasons including: enhanced quality of life, reduced adverse effects of chemotherapy, possibly enhanced effects of chemotherapeutic drugs, treatment of cancer-related symptoms and better clinical outcomes [68]. In a study of 1,455 primary breast cancer patients enrolled in the Shanghai Breast Cancer Study, ginseng use post-cancer diagnosis was positively associated with improved quality of life, with the strongest effects in the social and psychological wellbeing domains [69]. However, a later study of 4,149 women with primary breast cancer, who participated in the Shanghai Breast Cancer Survival Study, where ginseng use was assessed at 6- and 36- periods, showed no improved quality of life among the breast cancer survivors particularly in the physical, social and psychological domains [70]. The authors explained the variability in response to the design of the study and the different doses of ginseng used among breast cancer survivors.

In summary, these in vivo and in vitro studies provide evidence that ginseng and its active constituents such as Rh2 and Rg3 possess anti-cancer activity although the molecular mechanism is yet to be elucidated. Ginseng has potential as a chemotherapy adjunct, as the antitumor activity of ginseng is enhanced when used in combination with other conventional chemotherapy drugs. However, more clinical studies are needed, which may provide important evidence of the clinical benefits of ginseng.

## 3. Garlic (*Allium Sativum*)

### 3.1. The Bioactive Compounds of Garlic

Garlic (*Allium sativum*) belongs to the onion genus and is a bulbous perennial plant grown in mild climates, with a pungent and aroma taste which makes it quite useful as a flavoring agent. The major two subspecies of garlic are soft-necked garlic (*A. sativum var. sativum*) which comprises of creole garlic, silverskin garlic, antichoke garlic, and hard-neck garlic (A. *sativum var. ophioscorodon*) that includes rocambole garlic, purple stripe garlic and porcelain garlic [71]. Garlic has a high content of sulfur containing compounds which has been observed when fresh or crushed. These compounds include alliin (allyl 2-propenethiosulfinate or diallyl thiosulfinate), vinyldithiins, ajoene, S-allylcysteine, diallyl polysulfides, flavonoids and saponins [72]. Alliin, an amino acid and the major bioactive compound found in raw garlic homogenate or in aqueous extracts of garlic is converted to allicin by the enzyme alliinase. Allicin is an oily, slightly yellow organosulfur compound that contributes to the unique odor of garlic. When formed from Alliin, it is unstable and due to it being self-reactive, is quickly changed into a stable organosulfur compound such as diallyl disulfide [73]. Garlic also consists of compounds of steroidal and phenolic constituents, such as carbohydrates, fiber, proteins and trace elements like selenium [74]. Allyl sulfur compounds present in garlic are lipid soluble and include S-allylmercaptocysteine, diallyl trisulfide and diallyl disulfide [75].

### 3.2. In Vitro and in Vivo Studies of the Active Compounds of Ginseng and Their Anti-Cancer Effect

The United States Cancer Institute in 1990 reported in its Designer Food Program that garlic has potent food possessing cancer preventative properties [76]. Further, preclinical investigations provide convincing evidence that garlic and its organosulfur compounds inhibit carcinogen-induced tumors in various organs [77,78]. Over the last few decades, there have been numerous studies conducted in vitro and in vivo that have proposed possible anti-tumor effects of the bioactive constituents of garlic, mainly the allylsulfide derivatives in different preparations. Garlic derivatives such as S-allylcysteine (SAC) and S-allylmercaptocysteine (SAMC) have been described to moderate a number of molecular mechanisms in the initiation of cancer formation such as scavenging of free radicals, angiogenesis, DNA adduct formation, cell proliferation mutagenesis [79].

In vitro studies have shown that garlic and its derivatives, particularly diallyl disulfide, decrease the development of breast cancer in animals and suppress the progression of human breast cancer cells in culture [80,81]. The mechanism of action includes the induction of apoptosis, the regulation of cell-cycle arrest and stimulation of enzymes that are involved in the detoxification of carcinogens [82]. Diallyl disulfide was reported to synergize the outcome of the breast cancer suppressor eicosapentaenoic acid and antagonize the effects of the breast cancer enhancer linoleic acid, in breast cancer cell lines in culture [81].

There are other in vitro studies in which natural garlic and its derivatives in extracts are reported to inhibit the growth of human breast cancer cell lines, particularly MDA-MB-231 and MCF-7 in time and dose-dependent manner, by inducing the apoptosis and arrest of the cell cycle [82,83,84,85]. In a recent study that examined the anti-tumor efficacy of diallyl disulfide, the garlic derivative was found to impede the growth of breast cancer cells in vivo and in vitro using animal models [37]. The mechanism of action of diallyl disulfide involves the induction of apoptosis through the promotion of caspase-3 expression, upregulating the antioxidant enzymes superoxide dismutase and NQO1, and inhibiting the oxidative degradation of p53, an anti-tumor protein [37] (Table 1). Similarly, in an earlier study, diallyl trisulfide was reported to suppress the growth of non-tumorigenic MCF-12a mammary epithelial cells and MCF-7 human breast cancer cells by inducing apoptosis that was associated with the upregulation of p53 protein expression and increased pro-apoptotic Bax protein [86]. Garlic rich in selenium was found to be more potent than organosulfur analogues in inhibiting the growth of breast cancer cells in culture [81].

### 3.3. Clinical Studies with Garlic

There have been studies that have reported an inverse relationship between garlic intake and cancers of the stomach, lung and prostate [87,88]. However, there are fewer reports of the association between breast cancer and garlic intake. In a recent study of population-based, case-control study of 314 primary breast cancer cases, where dietary intake was assessed using a food frequency questionnaire, there was an inverse association between breast cancer and high consumption of garlic (OR = 0.51, 95% CI: 0.30–0.87) and moderate (OR = 0.59, 95% CI: 0.35–1.01) [89]. The authors suggest that high garlic consumption is protective against breast cancer in the Puerto Rican population [89] (Table 3).

In another case-control study of 345 patients with primary breast cancer in North-Eastern France where a self-administered dietary history questionnaire was assessed by accounting for established risk factors and total caloric intake, breast cancer risk was shown to be significantly reduced as consumption of garlic increased [90]. A meta-analysis of Swiss and Italian case-control studies which investigated the frequency of garlic use and cancer at different sites showed an odds ratio of 0.90 for breast cancer [91].

However, it is important to note that an analysis of cancer risk and garlic intake by using the United States Food and Drug Administration’s evidence-based review system showed no reliable evidence of an association between garlic intake and decreased risk of breast cancer, as well as gastric, lung and endometrial cancers [92].

In summary, these studies demonstrated that garlic and its derivatives such as diallyl disulfide display anti-cancer activities by retarding tumor growth and inducing apoptosis of human breast cancer cell lines and animal tumor models. Future clinical research should focus on the chemo-preventative properties of garlic, as helpful in targeting multiple pathways, as well as the molecular mechanisms involved, so as to give more credence to the prevention and treatment of breast cancers.

## 4. Black Cohosh (*Cimicifuga racemosa*)

### 4.1. The Bioactive Compounds of Black Cohosh

The herb black cohosh (*Actaea racemose*) formerly known as *Cimicifuga racemosa* is a flowering plant belonging to the family Ranunculaceae [98]. Black cohosh root consists of more than 42 triterpene glycosides, 11 phenolic acids, and more than 70 alkaloids and tannins. The triterpene glycosides such as actein, 23-epi-26-deoxyactein and cimiracemoside, as well as phenylpropanoids such as phenylpropanoid isoferulic acid are the major secondary compounds and biologically active components of black cohosh [99,100]. The alkaloids, tannins and flavonoids in the rhizome are regarded as possibly biologically active compounds [101]. Chemical research described approximately 15 polyphenolic components including fukiic acid, piscidic acid, caffeic acid and their derivatives [102]. Studies have also identified an isoflavone, formononetin, in black cohosh [103]. Remifemin, an extract of the rhizome is commercially formulated and along with other varieties of black cohosh though not standardized are available in the United States [104].

### 4.2. Clinical Studies with Black Cohosh

For many decades, black cohosh has been utilized in the treatment and management of dysmenorrhea, menopausal signs and symptoms such as hot flashes, and pre-menopausal discomfort [105]. Hot flashes are unexpected surges of sweats and hot skin due to decreased ovarian function as a consequence of breast cancer therapy or natural menopause. They arise less often in females experiencing perimenopause than menopause and there is evidence in the literature that among nonprescription therapies, black cohosh is a commonly used supplement to alleviate hot flashes [106]. Studies have reported that extracts from black cohosh lessens hot flashes as well as anxiety, insomnia and other peri-menopausal symptoms in patients with primary breast cancer [107,108]. The biological activity of black cohosh is due to the presence of various phytochemicals such as isoflavones which are estrogen-like substances and polyphenols [109].

It is claimed that phytoestrogens in black cohosh modulate central estrogen receptors close to the GnRH pulse generator of the hypothalamus through a negative feedback mechanism with a resulting estrogenic effect [110]. In addition to the estrogenic mechanism, it is proposed that black cohosh may act through a serotonergic mechanism that involves selective serotonin reuptake inhibitors [111]. There are also other proposed actions of black cohosh via other tissue-dependent mechanisms including anti-oxidative, anti-inflammatory and dopaminergic signaling where there is obstruction of ER-positive cell proliferation [112].

The treatment of breast cancer patients with tamoxifen frequently induces or exacerbates hot flashes and cause significant ill health in postmenopausal women. Hot flashes are common adverse reactions and can cause substantial morbidity in postmenopausal women during and after treatment. In the literature, there are reports of three randomized controlled trials that evaluated the effectiveness of black cohosh in reducing hot flashes in both pre- and postmenopausal breast cancer survivors. In one of these studies involving 136 breast cancer survivors administered with tamoxifen adjuvant therapy and black cohosh, there was satisfactory decrease in the severity and number of hot flashes [56] (Table 3).

There are two noteworthy prospective trials that have proposed the benefits of black cohosh on hot flashes. In a prospective observational trial involving 50 breast cancer patients treated with tamoxifen and subsequently administered with Remifemin (herbal preparation derived from black cohosh; one tablet twice daily (40 mg/d) for 6 months) there was a significant decrease in the severity of hot flashes with a baseline value of 17.6 to 13.6 using the Menopause Rating Scale (MRS-II) in the conclusion of the study [113]. A second prospective trial that investigated the effect of Remifemin (one capsule of 20 mg daily for 4 weeks) on hot flashes in 21 postmenopausal women with a history of breast cancer demonstrated a significant 56% decline in hot-flash score (95% CI: 40–71%) and mean number of hot flashes per day from a baseline value of 8.3 to 4.2 at the end of the study [114].

Two other studies demonstrated insignificant effects compared with placebo. In one study of 132 breast cancer patients in two 4-week crossover treatment phases, the mean hot flash scores decreased by 20% in those receiving black cohosh compared with a reduction of 27% for those on placebo [115]. In the second study of 89 breast cancer patients who had finished their treatment with tamoxifen, over a 60-day period post-treatment, there were decreases in the intensity and number of hot flashes. However, there were no statistically significant differences between those treated with black cohosh and placebo [116].

In summary, while these studies do not support the anti-cancer activity of black cohosh, there is evidence that supports its efficacy for the reduction of hot flashes, anxiety and other symptoms in patients with breast cancer. However, there is a need for more well-designed clinical trials to evaluate the therapeutic efficacy of black cohosh in breast cancer patients, especially those undergoing radiotherapy or chemotherapy.

## 5. Tumeric (*Curcuma longa*)

### 5.1. The Bioactive Compounds of Curcumin Longa

Curcuma *longa* is regarded as a perennial flowering plant belonging to the family Zingiberaceae. This rhizomatous, herbaceous plant is commonly known as turmeric possess roots which contain the major active ingredient curcuminoid [117]. Curcuminoids are natural polyphenol compounds and there are three types namely diferuloylmethane (curcumin I), desmetoxicurcumin (curcumin II) and bisdemetoxicurcumin (Curcumin III). The primary curcuminoid is diferuloylmethane that has the highest concentration (77%) and gives turmeric its yellow color [118]. Tumeric also contains sugars, resins, proteins as well as three main volatile oils (zingiberene, tumerone and atlatone) which possess pharmacological activity [119]. Curcumin is recognized as non-toxic and safe. Its therapeutic benefits are due to its anti-inflammatory and antioxidant effects [120]. However, the major concern with curcumin is its poor bioavailability due to low intestinal absorption coupled with rapid metabolism and elimination. The bioavailability of curcumin could be improved by developing innovative derivatives which block its metabolic pathway [121].

### 5.2. In Vitro Studies of the Anti-Cancer Effects of Curcumin

Studies investigating the anti-cancer activities of curcumin on breast cancer have reported on its mechanism of action, which involves several molecular targets [122]. These include: inhibition of cell proliferation, apoptosis, initiation of cell cycle arrest at the G2/M phase, upregulation of TIMP 1 and 4 expression, suppression of FABP5/PPARβ/δ pathway, inactivation of Akt/mTOR pathway and EGFR/PEGFR signaling pathway [122]. The molecular targets in the cell signal pathway that are modulated by curcumin include protein kinases B(Akt), AMP, β-catenin, ERK1/2, ERK5, EBPα, NF-κB, Nrf2, Notch-1, p38 MAPK, PPARγ, TGF-β1 and STAT3 [122].

The pro-inflammatory transcription factor nuclear factor-κB (NF-κB) modifies the expression of cytokines interleukin (IL)-2, interferon-γ (IFNγ) and IL-1 resulting in cell proliferation, survival and metastasis [123] (Table 1). According to a study by Liu et al., curcumin exerts its anti-tumor activity by the inhibition of cell proliferation of MDA-MB-231 and BT-483 breast cancer cells, and invasion via downregulation of NF-κB, transcription of matrix metalloproteinases (MMPs)-1 and cyclin D, a cell cycle regulatory protein [38] (Table 1). An in vitro study using MCF-7 breast cancer cells demonstrated that curcumin dose-dependently impedes the metastatic progression via suppression of urinary-type plasminogen activator (uPA) by downregulating NF-κB signaling pathways [124].

Signaling by autocrine growth hormone (GH) induced abnormal cell growth and differentiation, metastasis and resistance to chemotherapy drugs [125]. In a recent study, curcumin prohibited human GH triggered invasion and metastasis in T47D human breast cancer cells via downregulation of NF-κB signaling and miR-182-96-183 cluster expression [126]. In a similar study using MDA-MB-231, MCF-7 and MDA-MB-453 breast cancer cells, curcumin prohibited human GH triggered invasion and metastasis via suppression of NF-κB signaling, modulating cell survival and activating polyamine metabolism [127].

Abnormal activation of the Wnt/beta-catenin signaling pathway breast tumorigenesis and subsequent upregulation of beta-catenin driven downstream targets-c-Myc and cyclin D1 is associated with the development of breast cancer. Curcumin has been demonstrated to inhibit cell proliferation and induced apoptosis of MCF-7 and MDA-MB-231 breast cancer cells through the downregulation of the beta-catenin pathway [39] (Table 1). In addition to the Wnt/beta-catenin signaling pathway, the PI3K/Akt/mTOR pathway is activated in 30–40% of breast cancer cases and there is evidence of metastasis, angiogenesis, and therapy resistance [128]. In MDA-MB-231 breast cancer cells, curcumin dose-dependently reduced the expression of Akt protein as well as activate autophagy and suppressed the ubiquitin-proteasome pathway [129].

Apoptosis or programmed cell death is a physiological mechanism, characterized by specific morphological and biochemical changes such as cell shrinkage, chromatin condensation, protein cleavage, DNA breakdown and phagocytosis [130]. Curcumin exerted autophagy and induced apoptosis in MCF-7 breast cancer cells by downregulating the Bcl-2 signaling cascade and blocking the PI3K/Akt signaling pathway [131]. Moreover, it is suggested that curcumin has good prospects for treating HER-2-overexpressed breast cancer. In the BT-474 xenograft breast cancer model, it suppresses the HER-2 oncoprotein and downregulate the PI3K/Akt signal transduction, MAPK and NF-κB pathways [132].

MicroRNAs (miRNAs) are differentially expressed noncoding RNAs that control the expression of target genes. They regulate breast cancer cell initiation, proliferation and apoptosis [133]. It has been demonstrated that the anti-cancer effect of curcumin involves significant suppression of growth and apoptosis induction of MCF-7 breast cancer cells via the reduction of miR-21. The key molecular mechanism involved in the anti-cancer efficacy of curcumin is the inhibition of the miR-21/PTEN/Akt signaling pathway [134]. In another study using the MCF-7 human breast cancer cells, curcumin reversed the proliferative effects of bisphenol A (BPA) by downregulating oncogenic miR-19b and miR-19a and their downstream targets such as p53, p-Akt, PTEN and p-MDM2, and multiplying cell nuclear antigen [135]. The study also suggests that curcumin reversed the breast cancer progression by BPA by regulating the miR-19/PTEN/Akt/p53 pathway [135]. Furthermore, the incubation of curcumin with MCF-7 human breast cancer cells in vitro resulted in the downregulation of the apoptosis suppressor gene Bcl-2 by increased expression of miR-16 and miR-15 [136]. Interestingly, the putative anti-tumor actions of curcumin involve its interaction with a number of tumor-suppressive and oncogenic miRNAs such as miR-16, miR-15a, miR-34a and miR-181b which are upregulated, and miR-19b and miR-19a that are downregulated. These effects lead to the induction of apoptosis and G2/M cell cycle arrest, and the suppression of tumorigenesis and metastasis [137].

### 5.3. Effects of Curcumin in Combination with Anti-Cancer Drugs

Paclitaxel, a chemotherapeutic agent administered with curcumin has demonstrated a synergistic effect in inhibiting proliferation and inducing apoptosis in female Kunming mouse model and in MCF-7 human breast cancer cells [138]. The mechanism of action of the paclitaxel-curcumin combination involved a reduction in the expression of the regulatory protein Bcl-2 and decreased in the EGFR signaling blockade [138]. Similarly, paclitaxel and curcumin in corroborating their apoptotic effect decreased breast carcinogenesis by downregulating the expressions of Rho-A, p53, c-Ha-Ras and Bcl-2 in basal-like MDA-MB-231 human breast cancer cells [57] (Table 2). In an earlier metabolomic study conducted by Bayet-Robert and Morvan, curcumin alone or in association with docetaxel, a chemotherapy drug induces metabolic properties such as glucose utilization, lipid and glutathione metabolism in MDA-MB-231 and MCF-7 breast cancer cells [139].

5-fluorouracil (5-FU) is a fluorinated pyrimidine analog and a recognized chemotherapeutic agent for the treatment of breast cancer. It inhibits proliferation and initiates apoptosis by blocking the enzyme thymidylate synthase (TS), resulting in decreased synthesis of thymidine and less DNA replication [140]. The therapeutic efficacy of 5-FU is reduced due to resistance in breast cancer cells caused by overexpression of TS. Curcumin administration was found to sensitize MCF-7, SKBR3 and MDA-MB-231 breast cancer cells to 5-FU via reduction in the expression of TS thereby downregulating nuclear factor-κB [58] (Table 2). Moreover, curcumin increases the sensitivity of breast cancer cells to cisplatin, a potent antineoplastic drug by decreasing the expression of Flap endonuclease 1, a structure-specific nuclease that stimulates DNA replication and repair [141].

Recent studies have demonstrated that curcumin reverses the resistance caused by doxorubicin use via downregulation of the overexpression of ATP-binding cassette (ABC) transporters [142]. It also restores tamoxifen sensitivity via inhibition of chemo-resistant ATP-binding cassette (ABC) transporters in antiestrogen-resistant MCF-7/LCC2 and MCF-7/LCC9 breast cancer cell lines [143]. Finally, studies have reported a synergistic association of curcumin with other therapeutic agents such as carnosol, resveratrol, silibinin, mitomycin c and docosahexaneoic acid [144,145].

In summary, these studies demonstrated the chemo-preventative and therapeutic properties of curcumin in breast cancer. As a chemotherapeutic agent, it induces apoptosis, induces cell cycle arrest and its anti-proliferative effects involve the modulation of key transduction pathways and essential enzymes. Curcumin enhances the chemotherapeutic characteristics of conventional chemotherapy drugs such as paclitaxel and docetaxel. However, its limitations involve its application in vivo; as it has low aqueous solubility, narrow systemic distribution and undergoes significant biotransformation. Its efficacy and therapeutic potential could be improved by the use of liposome carriers and nanoparticles.

## 6. Camellia Sinenis (Green Tea)

### 6.1. The Bioactive Compounds of Green Tea

Green tea is produced from the fresh leaves (exposed to heat or hot steam) and buds of the evergreen plant Camellia sinensis [146]. Green tea consists of bioactive polyphenols, and extracts in powder or liquid forms differ in the proportion of 45.0–90.0% polyphenols and 0.4–10.0% caffeine. The polyphenols consist of flavonoids, flavandiols, flavanols and phenolic acids [147]. Catechins are a main class of flavonoids in the leaves of green tea and comprise 30–42% of the full dry weight of green tea. The catechins include epicatechin-3-gallate (ECG), epicatechin (EC), epigallocatechin-3-gallate (EGCG) and epigallocatechin (EGC), which represents 13.0%, 6.4%, 59.0% and 19.0% respectively [148]. The catechins present in the highest quantities are epigallocatechin-3-gallate, which accounts for 50–70% of the total quantity and is the most effective biologically active component of the leaves of green tea [149].

Flavones and flavonols present in green glycosides include: apigenin, quercetin, kaempferol and mycricetin [150]. In addition to polyphenols, green tea consists of amino acids such as aspartic acids, tryptophan, serine, threonine; carbohydrates such as fructose, cellulose, glucose and sucrose; minerals and trace elements such as magnesium, calcium, iron, selenium and aluminum; vitamins (E, C and B); alkaloids (3.0–4.0%) such as caffeine, theophylline, theobromide and methylxanthines [151].

The consumption of green tea has been associated with the prevention of various kinds of cancers, including breast, colon, esophagus, kidney, lung, mouth, pancreas, stomach and small intestine due to its antioxidant, anti-mutagenic and chemo-preventative effects [152]. In addition, clinical trials and epidemiological studies demonstrated that green tea may reduce the risk of many chronic non-communicable diseases [153]. The efficacy of green tea has been attributed to epigallocatechin-3-gallate (EGCG) [154].

### 6.2. In Vivo and Clinical Studies of the Anti-Cancer Effects of Green Tea

Preclinical studies have demonstrated that green tea or its components (mainly epigallocatechin-3-gallate) display chemo-preventative effects in the development of breast cancer [93,94,155]. A number of studies evaluated whether green tea consumption or its constituents could be effective in reducing breast cancer risk [94,155,156]. In the sister study, utilizing data of 45,744 United States and Puerto Rica females, drinking five cups or more green tea per week may be related to a reduction in breast cancer risk [156]. Similarly in a case-control study of 1009 female patients with primary breast cancer and their 1009 age-matched controls in Southeast Asia, consistent consumption of dried green tea leaves offers protection against breast cancer (OR = 0.61, 95% CI: 0.48–0.78; *p* < 0.01 for 500–749 g per annum) [93] (Table 3). Moreover, in a study of 472 female breast cancer patients with stage I, II and III breast cancer, the consumption of five or more cups per day gave a relative risk of recurrence of 0.564 (95% CI: 0.35–0.91) and prior use before diagnosis was significantly associated with better prognosis of stage I and II [94] (Table 3). In addition, in a recent observational study of 1,551 breast cancer patients, better progression-free and survival was seen in those who regularly consumed green tea (HR 0.30; 95% CI: 0.11–0.84) particularly those females with normal lipids [155].

Conversely, the evidence from epidemiological studies is not consistent. In a hospital-based case-control study of 439 hospital controls and 434 breast cancer cases, regular consumption of tea was significantly associated with slightly non-significant increased risk (OR = 0.62, 95%CI: 0.40–0.97) but not overall risk [157]. In addition, a prospective study of 1,268 incident cases of breast cancer with a follow-up of 12 years, green tea consumption of ≥4 cups/day was not related to breast cancer risk among African American women [158].

As the overall results from prospective and case-control studies conflict, partly due to relatively small numbers, researchers have conducted systematic reviews and meta-analyses of publications in this area of study. In a systematic and meta-analysis of three case-control studies by Wu and Butler, there was a 30% (95% CI: 0.61–0.79) decreased breast cancer risk for consistent green tea consumption among patients [159]. Similarly, a meta-analysis 163,810 breast cancer patients in eight cohort studies and five case-control studies showed an inverse statistically significant relationship between breast cancer risk and green tea consumption (OR = 0.85, 95% CI: 0.80–0.92, *p* = 0.0001; [160].

Other reported systematic and meta-analysis reviews of studies showed an inverse relationship between increased green tea consumption (>3 cups/day) and risk of breast cancer recurrence [161]; no significant relationship between consumption of ≥ cups of green tea/day and developing breast cancer in cohort studies (RR = 0.89, 95% CI: 0.71–1.10, *p* > 0.05) [162] and the analysis of nine case-control studies, four cohort studies revealed that green tea consumption may not reduce breast cancer risk (overall OR = 0.81, 95% CI: 0.66–0.98, *p* = 0.031) [163].

As the findings from epidemiological studies vary, a biomarker approach is suggested as a better way of assessing the relationship between breast cancer risk and green tea consumption, particularly measuring the effects of specific tea catechins [164,165]. In a cohort prospective study conducted in China of 353 breast cancer cases and 701 matched nested controls, urinary tea polyphenols (4′-methyl-epigallocatechin, epicatechin and epigallocatechin) and their metabolites as well as flavonols such as kaempferol and quercetin were determined by mass spectrometry. The majority of the patients regularly consumed at least one cup of green tea, and they reported an inverse relationship between breast cancer risk and urinary concentration of epicatechin (OR = 0.61, 95% CI: 0.39–0.88) for the intermediate excretion range [164]. In another prospective cohort study of 144 patients with breast cancer and 288 matched controls, plasma concentrations of tea catechins such as epicatechin-3-gallate, epigallocatechin, epicatechin and EGCG were determined. There was no overall statistically significant relationship between breast cancer risk and the plasma levels of the tea catechins [165].

Mammographic density denotes the percentage of dense tissue of the whole breast and is a recognized risk factor of breast cancer [166]. In a cross-sectional study of 3,315 breast cancer patients, those who daily consume green tea (particularly postmenopausal women) demonstrated a statistically significant reduction in mammographic density percentage (19.5%) than non-tea users (21.7%; *p* < 0.05) after correction for numerous possible confounders [167]. In a latter study that was a randomized, double-blinded, placebo-controlled phase II clinical trial, 1,075 healthy postmenopausal women were supplemented daily with four decaffeinated green tea capsules (1315 mg total catechins of which 843 mg is epigallocatechin-3-gallate) for 12 months. There was a significant reduction in the change percent mammographic density (PMD) by 4.40% compared with 1.02% PMD elevation for pre-menopausal women, but not for their postmenopausal counterparts [168]. The authors suggested that further exploration of the possible chemo-preventative effect of the consumption of green tea on the risk of breast cancer in pre-menopausal women is merited [168].

In in vitro studies, the enzyme catechol-O-methyltransferase (COMT) is inhibited by epigallocatechin-3-gallate. Polymorphism in the COMT gene resulted in a 40% decrease in enzymatic activity and this may modify the relationship between breast cancer risk and green tea consumption [169]. In a case-control study of 589 incident cases and 563 population-based controls conducted among Asian-American women, Wu et al. observed that the consumption of green tea was significantly related to decreased breast cancer risk (adjusted OR = 0.48; 95% CI: 0.29–0.77) among those who possess low activity COMT allele compared with nondrinkers. Conversely, no relationship was observed between green tea consumption and breast cancer risk among those who possess high activity COMT allele compared with nondrinkers (adjusted OR = 1.02; 95% CI: 0.66–1.60) [170]. In addition, a population-based case-control study of 3454 incident breast cancer cases and 3474 controls (aged 20–74 years) conducted in a Chinese population showed that the COMT rs4680 genotypes did not have any altering effect on the relationship between breast cancer risk and green tea consumption [171]. The association between regular green tea intake and reduced breast cancer risk is weak (OR = 0.88; 95% CI: 0.79–0.98) compared with nondrinkers [171]. Therefore, additional studies are warranted to resolve these varying findings.

### 6.3. Effects of Epigallocatechin-3-Gallate in Combination with Anti-Cancer Drugs

Green tea and EGCG possess potent anti-cancer and antioxidant properties, and studies have investigated any synergistic relationship with chemotherapeutic agents [59,172,173]. 5-aza-2-deoxycytidine is a demethylating agent that improves the susceptibility of breast cancer cells to anti-cancer drugs. The combination of EGCG and 5-aza-2-deoxycytidine on MCF-7 and MDA-MB-231 human breast cancer cell lines caused significant inhibition of cell proliferation compared to individual treatment and decreased toxicity of the demethylating agent [172]. Similarly, MCF-7 and MDA-MB-231 breast cancer cells treated with EGCG and quercetin, as well as tamoxifen resulted in reduced cell proliferation [59]. Another in vivo study reported that the combination of Suberoylanilide hydroxamic acid (SAHA) and EGCG inhibits growth and proliferation of triple-negative breast cancer cell lines via the modulating of the expression of miR-221/222 and tumor suppressors, PTEN and p27 [173]. Moreover, the combination of EGCG and sunitinib in MCF-7, H460, and H1975 breast cancer cell lines resulted in greater shrinkage of tumor than with the drug alone via suppression of the IRS/MAPK signaling pathway, induced by EGCG [174]. Together, the findings of a synergistic relationship of EGCG with anti-cancer agents reported in these studies represent a promising approach for the treatment of breast cancer.

In summary, in vivo and in vitro studies demonstrated the anti-cancer effects of green tea and its synergistic relationship with conventional chemotherapeutic agents. The mechanism of action including the modulation of different intracellular signaling pathways. The chemo-preventative and chemotherapeutic agent in green tea is the polyphenol epigallocatechin-3-gallate which has a key role in stimulating apoptosis, a critical aspect of breast cancer prevention. Polyphenols and other components of green tea may exert beneficial effects as they suppress breast cancer development particularly in premenopausal women and prevent recurrence. However, epidemiological studies using green tea are inconclusive and the mechanisms by which green tea consumption may influence breast cancer risk in humans remain unclear.

## 7. Echinacea

### 7.1. Species of Echinacea and Their Bioactive Compounds

Echinacea is a traditional herbal and medicinal plant that belongs to the family Asteraceae. This herbaceous perennial aromatic plant is endemic to North America but is also cultivated in Europe [175]. There are nine species of Echinacea, three of which are commonly used as phyto-therapeutic products. These are *Echinacea angustifolia*, *Echinacea purpurea* and *Echinacea pallida*. The roots and rhizomes of the three species and the flowering parts of *Echinacea purpurea* are used for medicinal purposes that are present in 80% of the commercial products of Echinacea [176]. High-pressure liquid chromatography has been used to identify and isolate the chemical constituents of Echinacea. The components that have been recognized are caffeic acid, chicoric acid, cynarin, chlorogenic acid, echinacin, essential oils, isobutyl amides, fatty acids, flavonoids, isotussilagine, polyenes terpenoids, polyacetylenes and phytosterols [176]. The active ingredients of Echinacea are alkyl amides, chicoric acid, essential oils, flavonoids, polyacetylenes and polysaccharides [176]. The aerials and roots of *Echinacea purpurea* contain chicoric acid, caftaric acid and chlorogenic acid. On the other hand, the root of *Echinacea pallida* contains chicoric acid, caftaric acid and echinacoside [159]. Furthermore, the aerial and roots of *Echinacea angustifolia* contain cynarin, chicoric, acid, echinacoside, polyacetylenes and polysaccharides [177]. Echinacea is a common herbal supplement that is rich in flavonoids, extensively used for its therapeutic properties including anti-inflammatory, antioxidant and immune-stimulant properties [178].

### 7.2. In Vitro and Clinical Studies of Echinacea and Drug-Herbal Interaction

Echinacea is a commonly used herbal supplement among cancer patients, and in a study of 318 patients, it was the most popular herbal medicine by 21% of respondents [179]. There are few studies that have examined the usage of Echinacea among patients with breast cancer [180,181]. Ma et al. reported that herbal remedies including Echinacea, ginko biloba and herbal teas were associated with worse physical component scores for health-related quality of life among breast cancer survivors [180]. The Black Women’s Health Study documented the increased use of herbs such as Echinacea among breast cancer survivors and the possible interaction with adjuvant therapies such as anastrazole and tamoxifen [181].

The potential of Echinacea for anti-tumor therapy was investigated using in vitro studies. Extracts of *Echinacea purpurea* was found to significantly reduce the growth of BT-549 mammalian breast cancer cell line, and its effect was more potent than *Echinacea Pallida* [40] (Table 1). A latter study showed that pentadeca-(8Z, 13Z)-dien-11-yn-Z-one, a constituent of the root of *Echinacea Pallida* decreased proliferation of MCF-7 breast cancer cell line [182].

Echinacea preparations are among the popular herbal medicinal products, but they have been shown to induce inhibition of cytochrome P450 3A4 (CYP 3A4) isoenzyme as well as other isoforms CYP 2C19, CYP 2D9, and CYP IA2 both in humans and in vitro [183]. Doxorubicin is an anti-cancer drug that is used for the management of metastatic and locally advanced breast cancer. It is expansively metabolized by hepatic CYP3A4, and induction of this enzyme by *Echinacea purpurea* and *Echinacea Pallida* may decrease its efficacy, reduce the plasma levels and possibly cause an under-treatment for patients receiving this anti-cancer drug [184]. Huntimer and colleagues reported that constituents of the roots of *Echinacea angustifolia (ethyl acetate fraction and chicoric acid) and* doxorubicin increased cell growth of MCF-7 breast cancer cells and could interfere with the efficacy of the anti-cancer drug [185]. An in vitro study involving the treatment of MCF-7 breast cancer cells with hexane fractions of *Echinacea purpurea* reported a reduction in proliferation, and cynarin from the roots of *Echinacea angustifolia* also showed anti-proliferative activity. The authors also posited that cynarin enhanced the cytotoxic activity of doxorubicin against MCF-7 breast cancer cells [60]. Based on the potential for *Echinacea*-doxorubicin interaction, it is suggested that the former should be monitored more closely. There is also a report of a safe dose of an extract of *Echinacea purpurea* that did not interact with docetaxel as well as a recommended schedule proposed by Goey and colleagues [60] (Table 2).

In summary, Echinacea is becoming a very popular herbal medicine and there are studies that have reported the use of Echinacea among breast cancer patients. A few in vivo and in vitro studies have attested to the anti-cancer properties of the components of *Echinacea* in different extracts. However, its inhibition of cytochrome P450 enzymes both in vitro and in humans limits its therapeutic efficacy.

## 8. Arctium (Burdock)

### 8.1. The Bioactive Compounds of Burdock

The species of the genus Arctium commonly known as burdock consist of herbaceous perennial plants with stout and erect stems and roots, and hairy leaves that grows by streams and roadsides [186]. There are 18 documented species of Arctium of which three are quite common particularly in central Europe. They are *Arctium lappa* (greater burdock), *Arctium tomentosum* (wooly burdock) and *Arctium minus* (lesser burdock) [187].

Arctium *lappa* is a traditional medicine in China and other parts of the world. Its roots is used in Europe for the management of dermatological and blood disorders [188]; its leaves are used as an anti-inflammatory agent in traditional medicine to treat gastrointestinal disorders in Brazil [189]. Both fruits and roots are used to treat diabetes mellitus in Asian countries [190]. The European Medicines Agency (EMA) recommends the roots of *Arctium tomentosum* and of *Arctium lappa* as adjunct therapy for seborrheic skin conditions and urinary tract infections [191].

The Arctium genus comprised of 200 non-volatile compounds including lactones, lignans, flavonoids, quinic acids, phenolics, polyacetylenes, terpenoids and polyssacharides [192]. Lignans are the biologically active components of the Arctium genus and include mainly arctigenin, a dietary phytoestrogen and arctiin its glycoside present in fruits, leaves, seeds and roots of *Arctium tomentosum* and *Arctium* lappa [193,194]. Flavonoids present in the Arctium genus include flavones, flavonols and their glycosides. Flavonoids such as isoquercetin and rutin, which are the two main constituents, and minor ones including quercimeritrin, quercetin, quercitrin, astragalin and quercetin-3-O-rhamnoside, have been reported to be present in leaves, fruits, seeds and roots of *Arctium lappa* [194,195]. Arctigenin is widely used in traditional medicine and numerous studies have reported the therapeutic potential of this compound as an immune modulator, anti-inflammatory and antitumor agent [196,197]. There has been significant interest in the antitumor activity of arctigenin, particularly in in vitro studies using several human cancer cell lines such as the lung, stomach, intestine, ovaries and breast [41,198,199].

### 8.2. In Vitro Studies of the Antitumor Activities of Arctium Lappa

Studies that investigated the antitumor activities of *Arctium lappa* involve mainly in vitro studies using human cell lines. Lou et al. examined the effects of arctigenin from *Arctium lappa* on tumor invasion and migration of MDA-MB-231 breast cancer cells. Arctigenin significantly inhibited the metastasis of the breast cancer cells although the cytotoxic effect on the cells was not significant [197]. The mechanism of action of arctigenin was the downregulation of the expression of matrix metallo-proteinases, MMP-2 and MMP-9 which has a role in metastasis, as well as heparanase expression [197]. In an earlier study, arctigenin from *Arctium lappa* inhibited cell growth of MDA-MB-231 breast cancer cells by inducing apoptosis in vivo and in vitro. The mechanism of apoptosis involves activation of the ROS/p38 MAPK pathway which subsequently induce mitochondrial caspase-independent pathways with increased Bax/Bcl-2 ratio [41] (Table 1). In another study, arctigen exerted anti-metastatic effects on both MCF-7 and MDA-MB-231 human breast cancer cell lines by inhibiting the NF-κB, Akt/MAPK signaling pathways, and MMP-9 [42] (Table 1). Moreover, the dichloromethanic extract of *Arctium lappa* roots which contain chlorogenic acid, quercetin, caffeic acid and arctigenin compounds exhibited anti-proliferative activity against MCF-7 breast cancer cells [200].

### 8.3. Effects of Arctium Lappa in Combination with Anti-Cancer Drugs

Doxorubicin is an effective chemotherapy drug. However, it causes a dose and cumulative-dependent cardio-toxicity that limits its efficacy. Ghafari et al. compared the anti-proliferative effects of doxorubicin and *Arctium lappa* on MCF-7 and MDA-MB-231 breast cancer cell lines. The extract from the roots of *Arctium lappa* decreased cell viability and induced apoptosis on both cell lines in a time and dose-dependent manner comparable to doxorubicin [61] (Table 2). In another study, arctigenin decreased the proliferation and inhibited apoptosis of triple-negative breast cancer cells via downregulation of signal transducers and activators of transcription (STATs) particularly STAT3 [201]. It is noteworthy that arctigenin augmented the cytotoxicity induced by taxotere on triple-negative breast cancer cells [201].

## 9. Flaxseed (*Linum usitatissimum*)

### 9.1. The Bioactive Compounds of Flaxseed

Flaxseed (*Linum usitatissimum*), also commonly known as linseed, is a member of the *Linaceae* family and is cultivated mainly in Asia, Europe and in the Mediterranean region. Flaxseed is one of the oldest crops and consists of two species, brown and yellow (or golden) with a comparable number of short-chain ω-3 fatty acids and nutritional characteristics [202]. Flaxseed is a main plant source of essential fatty acids and its physico-composition includes: lignans, minerals such as magnesium, phosphorous and calcium, proteins such as globulins (linin and conlinin) and glutelin present in ratios of 80% to 20%, insoluble (cellulose and lignin) and soluble (mucilage gums) dietary fibers, and soluble polysaccharides and vitamins (A, C and E) [203].

Flaxseed comprises approximately 800 times more lignans than other plants. They are bioactive, non-nutritional and phenolic compounds that are phytoestrogens and comprised predominantly of secoisolariciresinol diglucoside (SDG) (294–700 mg/100 g) which makes up approximately 95% of the lignin content with the remaining 5% consisting of lariciresinol (3.04 mg/100 g), pinoresinol (3.32 mg/100 g) and matairesinol (0.55 mg/100 g) [204]. Flaxseed has dietary characteristics and its oil consists of lipids such as α-linolenic acid (ω-3 fatty acid) the main fatty acids (39.00–60.42%) along with linoleic, oleic, stearic and palmate acids [205]. Flaxseed oils are also comprised of polyunsaturated fatty acid (73%), mono-saturated fatty acids (18%) and saturated fatty acids (9%) [206]. The possible health benefits of flaxseed include nutrition due to the high content of α-linolenic acid, richness in both insoluble and soluble fibers, and lignans which have both estrogenic and antioxidant properties [207].

### 9.2. Experimental In Vitro Studies of the Antitumor Activities of Flaxseed

α-linolenic acid can be metabolized into eicosapentaenoic acid (EPA) (ω-3) and docosahexaenoic acid (DHA) (ω-3) and all three ω-3 fatty acids have been widely described in numerous conditions including: diabetes mellitus, neurological disorders, atherosclerosis, hypertension and cardiovascular disease [208]. Experimental studies have investigated the anti-tumorigenic effect of flaxseed and some of these involved animals, where mice were injected with breast tumor cells. Chen et al. investigated the effects of dietary flaxseed on the growth of human breast tumor, and possible mechanism(s) of action using athymic mice inoculated with human estrogen receptor (ER) positive breast cancer cells (MCF-7). There were significant inhibition of cell proliferation and induced apoptosis via reduced mRNA expressions of cyclin D1, epidermal growth factor receptor and Bcl-2 [43] (Table 1). Earlier, the same research group reported a 45% reduction in tumor growth rate in a nude mice model injected with MDA-MB-435 treated with 10% flaxseed.

Immuno-histochemical study revealed decreased expression of both epidermal growth factor receptor and insulin-like growth factor I [209]. In another study, flaxseed and its lignan, enterolactone, counteracted angiogenesis and ex-induced growth in mice inoculated with MCF-7 human breast cancer cells. The in vivo findings were confirmed in vitro as flaxseed and its lignans inhibited the secretion of vascular endothelial growth factor, which is an effective potent stimulator of angiogenesis [210]. Conversely, secoisolariciresinol diglucoside, a lignin present in flaxseed, did not reduce breast tumor growth nor induce apoptosis in athymic mice [211].

### 9.3. Effects of Flaxseed in Combination with Anti-Cancer Drugs

Tamoxifen is a well-recognized adjuvant treatment for women who are ER-positive and also for metastatic breast cancer [212]. The side effects of tamoxifen include hot flashes and many patients with breast cancer use phytoestrogen-rich foods such as flaxseed and soy to diminish the symptoms and also to enhance the effect of the drug [213]. There are studies that have investigated whether flaxseed augments or interferes with the effect of tamoxifen. In an in vivo study conducted by Chen et al. with athymic mice inoculated with MCF-7 breast cancer cells and implanted with an E2 pellet, flaxseed inhibited the growth of the tumor size by 74%, while both tamoxifen and flaxseed caused tumor regression by over 53%. It was observed that flaxseed at both high and low E2 levels augment the tumor-inhibited effects of tamoxifen [62] (Table 2). In another study by the same research group, long-term treatment of athymic mice with combined tamoxifen and 10% flaxseed reduced the tumor size by 55%, due to the induction of apoptosis and decreased cell proliferation. The mechanisms of action involves the downregulation of the expressions of estrogen receptor alpha, human epidermal growth factor receptor 2 and cyclin D1, as well as signal transduction pathways [214]. There was a similar finding by Saggar et al., where secoisolariciresinol diglucoside and flaxseed oil decreased the growth of tamoxifen-treated tumors via decreasing the expressions of genes and proteins involved in the estrogen receptor- and growth factor-mediated signaling pathways [215]. Further, flaxseed oil enhanced the reducing effects of trastuzumab on HER2-overexpressing tumor (BT-474) growth via lowering the phosphorylated/total phosphorylated expression of MAPK and Akt proteins [63] (Table 2).

### 9.4. Clinical Studies of the Anti-Cancer Effects of Flaxseed

There are observational studies that have suggested that the consumption of flaxseed can reduce the risk of breast cancer. In the Ontario Women’s Diet and Health Study of 2999 breast cancer cases and 3,370 healthy control, flaxseed consumption was associated with a significant decrease in breast cancer risk (OR = 0.77, 95% CI: 0.67–0.89) [95] (Table 3). In a randomized double-blind placebo-controlled clinical trial of postmenopausal women newly diagnosed with breast cancer, dietary flaxseed reduced tumor growth. This was concomitant with the downregulation of c-erbB2 expression and reduced Ki-67 labeling index, a marker of tumor cell proliferation [96] (Table 3). Conversely, isoflavones or lignans from flaxseed did not significantly reduce breast cancer risk of breast cancer cases in the Ontario Cancer Registry [216].

There have been a number of systematic reviews of the efficiency of flaxseed or its lignans in reducing breast cancer risk. In a review of 10 studies by Fower et al., flaxseed or secoisolariciresinol diglycoside consumed daily significantly decreased breast cancer risk via increased tumor apoptotic index, and reduced cell proliferation and HER2 expression [217]. Another meta-analysis of 21 studies (11 prospective cohort studies and 10 case-control studies) reported an association between high lignin intake and reduced breast cancer risk in postmenopausal women [218]. Similarly, in three separate meta-analyses, high levels of lignan or enterolignan intake significantly lower the risk of breast cancer [219].

There are components of flaxseed such as α-linolenic acid and secoisolariciresinol diglycoside, enterolactone, enterodiol and ω-3 polyunsaturated fatty acids that were studied in clinical studies for their beneficial anti-oncotic action. It is reported that higher ω-3 polyunsaturated fatty acids that possess anti-inflammatory properties may reduce breast cancer risk [220]. In a population-based case-control study of 3024 cases and 3420 controls, dietary phytoestrogen intake (isoflavones and lignans) during adolescence was concomitant with decreased breast cancer risk [221]. In an earlier conducted prospective cohort study of 58,049 postmenopausal French women followed for 7.7 years, those with total lignan intake (pinoresinol, lariciresinol, secoisolariciresinol, and matairesinol) greater than 1395 micrograms/day had a significant reduction on breast cancer risk. It is noted that the favorable effects of the lignans were limited to breast cancer cases who were progesterone receptor-positive and ER+ [222]. Additionally, postmenopausal breast cancer patients with high levels of enterolactone had significantly reduced the risk of death, particularly those who were estrogen receptor-negative [223]. Conversely, a prospective cohort study suggests that high levels of enterolactone may not protect women from developing breast cancer [224]. Therefore, randomized control trials are needed to fully establish the association between flaxseed and its components, and breast cancer risk.

In summary, experiments involving culture cells and tumor animal models showed that flaxseed exhibited anti-cancer properties as there was a reduction of tumor growth and increased apoptosis. There is a synergistic relationship between flaxseed and tamoxifen as the herb increases or maintains the efficacy of the chemotherapy drug. Clinical studies have concluded that flaxseed has the potential to decrease the risk, tumor growth and size in breast cancer patients. However, more clinical trials are necessary to authenticate the benefits of flaxseed on breast cancer therapy.

## 10. Black Cumin (*Nigella sativa*)

### 10.1. The Bioactive Compounds of Nigella sativa

*Nigella sativa*, also well-known as black cumin is a medicinal herb and an annual flowering plant belonging to the family Ranunculaceae [225]. Numerous active ingredients in *Nigella sativa* are present in the seeds. The seeds of Nigella sativa comprise fats (28.5%), proteins (26.7%), carbohydrates (24.9%), total ash (4.8%) and crude fibre (8.4%) [226,227]. In examining the fat components in the seeds of *Nigella sativa*, oils constitutes 32–40% of the total composition, unsaturated fatty acid such as linoleic acid is present in high concentrations (50–60%). Other unsaturated fatty acids include dihomolinoleic acid (10%), eicosadienoic acid (3%) and oleic acid (20%) and the notable presence of saturated fatty acids such as stearic and palmitic stearic acids that comprise approximately 30 per cent [228,229]. There are over 100 bioactive compounds present in the seeds of *Nigella sativa*; four pharmacologically significant ones are thymol, thymohydroquinone, thymoquinone and dithymoquinone [229].

Thymoquinone is the main biologically active compound of *Nigella sativa* and constitute 30–48%, followed by others such as p-cymene, dithymoquinone, thymohydroquinone (7–15%), carvacrol (6–12%), 4-terpineol (2–7%), t-anethole (1–4%), sesquiterpene longifolene (1–8%) and the thymol, α-pinene [230]. Thymoquinone being the main component of the essential oil in the seeds of *Nigella sativa*, along the seeds are utilized for medicinal purposes and possesses antidiabetic, anti-cancer, anti-inflammatory, hepato-protective, anti-microbial, immunomodulatory and antioxidant properties [231,232].

### 10.2. Thymoquinone’s Anti-Cancer Effects In Vitro and In Vivo Animal Models

Apoptosis is a highly selective and ordered process of programmed cell death and occurs both in physiological and pathological conditions [233]. There are a number of studies that have shown that thymoquinone inhibits tumorigenesis via a number of molecular mechanisms, and there is evidence that in in vitro applications, it induces apoptosis in several breast cancer cell lines [234,235]. Thymoquinone has been shown to have antineoplastic activity and [44] (Table 1). Rajput et al. [44] investigated its mechanism of action relating to PI3K/Akt signaling and downstream targets with subsequent apoptosis in cancer cells. Thymoquinone induces apoptosis in T-47D and MDA-MB-468 breast cancer cells by promoting G(1) phase arrest via translation upregulation of procaspase-3, Bax and cytoplasmic cytochrome 3, inhibition of cyclin D1 and cyclin E, and PARP cleavage alongside downregulation of the gene expression of survivin, Bcl-2 and Bcl-xL [44] (Table 1). In a later study, thymoquinone induces apoptosis in MCF-7 breast cancer cell line through the upregulation of the expression of tumor suppressor gene p53 in a time-dependent manner [45] (Table 1). Similarly, another study that investigated the efficacy of long-term in vitro treatment with thymoquinone showed sustained inhibition of proliferation of MCF-7 human breast cancer cell lines due to S phase and G2 phase arrest [236]. There is also evidence that methanolic extract of *Nigella sativa* (at doses of 2.5–5 μg/mL) suppresses the proliferation of human breast cancer MDA-MB-231 cells via the induction of apoptosis [237]. Methanolic extract of *Nigella sativa* seeds also induced apoptosis in human breast cancer MCF-7 cells via both the caspase and p53 pathways [238]. Notably, the mechanism of the anti-proliferative effect of thymoquinone on MDA-MB-231 breast cancer cells involves the modulation of the PPAR-γ activation pathway. Thymoquinone elevates PPAR-γ activity and downregulates the expression of genes for survivin, Bcl-xL and Bcl-2 in MDA-MB-231 breast cancer cells [239].

Metastatic breast cancer is advanced or stage IV breast cancer that has spread more likely to the liver, bones, lungs or brain [240]. There are studies that have investigated the potential of thymoquinone from *Nigella sativa* to suppress tumor growth and metastasis of breast cancer in in vitro and animal models [241,242]. Shanmugam et al. investigated the possible effect of thymoquinone from the seeds of Nigella sativa on the regulation and expression of chemokine receptor type 4 (CXCR4) in MDA-MB-231 triple-negative breast cancer cells [46]. Thymoquinone downregulated the expressions of CXCR4 in breast cancer cells in a time- and dose-dependent manner, demonstrating its anti-metastatic effect [46] (Table 1). In an earlier study, seed extracts from *Nigella sativa* injected into the tumor site of DBA2/P815 (H2d) mouse decrease tumor volume, inhibited the development of liver metastasis and improved survival [243]. Likewise, thymoquinone treatment inhibited the development and metastasis of cell-derived xenograft tumors in mice via reduced activity and mRNA expression of the transcription factor TWIST1. This resulted in reduced migration and invasion of breast cancer cells [244]. Interestingly, supercritical carbon dioxide extracts of the seeds of *Nigella sativa* exhibited anti-metastatic and pro-apoptotic properties by inhibiting migration and invasion of estrogen-dependent human breast cancer cells (MCF-7) sub-cytotoxic concentrations [245]. In a recent study involving MDA-MB-231 breast cancer cells, thymoquinone treatment induced the expression of tristetraprolin, a tumor suppressor gene. This resulted in inhibition of cell growth and proliferation, and metastasis via activation of the Raf-MEK-ERK pathway and resulting destabilization of MUC4 mRNA [246].

There are few studies that have investigated the anti-cancer effect of thymoquinone from the seeds oil of *Nigella sativa* in animal models [247,248]. Linjawi et al. investigated the effects of the oil of black cumin seed (*Nigella sativa*) and thymoquinone on serum tumor markers such as lactate dehydrogenase (LDH) and malondialdehyde as well as and liver enzymes including aspartate aminotransferase (AST) and alkaline phosphatase (ALP) in DMBA-induced breast cancer in female rats [249]. Black cumin seed (*Nigella sativa*) and thymoquinone exercised a protective effect as they reduced the breast carcinogens and decreased the gene expressions of Id-1, Brac2, Bra1 and p53 mutations [249]. In another study, in vivo administration of thymoquinone (20 and 100 mg/kg) significantly decreased the progression of MDA-MB-231 tumors and inhibited the eukaryotic elongation factor 2 kinase (eEF-2K) in an orthotopic tumor mouse model of triple-negative breast cancer [250].

Thymoquinone is hydrophobic and lipophilic and these two properties limit its oral bioavailability and efficacy. The pro-apoptotic and anti-proliferative effects of thymoquinone in the breast tumor xenograft mouse model are intermediated through reactive oxygen species and p38 phosphorylation. The mechanism of action of thymoquinone involves downregulation of the protein expression of anti-apoptotic genes, such as surviving, Bcl-2, XIAP and Bsl-xL [251]. The anti-cancer activities of thymoquinone in BALB/c mice were enhanced by its encapsulation in a nanostructured lipid carrier [252]. In addition to its anti-cancer properties, thymoquinone significantly demonstrates hepato-protective effects. Thymoquinone inhibited tamoxifen-induced hepatic damage in female rats with less glutathione depletion and lipid peroxide accumulation [253].

### 10.3. Pharmacokinetic Characteristics of Thymoquinone and Its Combination with Other Chemotherapeutic Drugs

Thymoquinone plays an important role in chemoprevention as it modulates tumor suppressor genes in its anti-apoptotic activity [254]. However, its use is limited due to its low bioavailability and hydrophobic properties as it has poor aqueous solubility [255]. Studies that has examined its pharmacokinetic properties reported that it is slowly absorbed in the body and rapidly eliminated [256,257]. The delivery of thymoquinone is enhanced by the use of nanoparticles known as nanostructured lipid carriers which bring about improved drug reactivity [258,259]. Bhattacharya et al. produced a thymoquinone-encapsulated nanoparticle utilizing hydrophilic biodegradable polymers such as polyethyleneglycol aimed at improving the drug’s aqueous solubility and systemic bioavailability. Polyethyleneglycol-thymoquinone-encapsulated nanoparticle (PEG4000-TQ-Nps) induced apoptosis and reduced migration of breast cancer cells in vivo via disruption of cytoskeletal actin polymerization caused by the increased expression of miR-34a and downregulation of Rac1 expression [259]. Similarly, thymoquinone-loaded nanostructured lipid carrier (TQ-NLC) exhibited anti-proliferative activity towards MDA-MB-231 cells in a dose-dependent manner. TQ-NLC caused cell shrinkage with an increase in apoptotic cell population and cell cycle arrest of the MDA-MB-231 cell line [260]. Notably, TQ-NLC along with thymoquinone inhibited lung metastasis in 4T1 tumor-bearing female BALB/c mice by downregulating the expression of MMP-2 and activating the intrinsic apoptotic pathway in the cancer tumor cells [261]. In another study that utilized nanogel-based nanoparticle to improve the efficacy of thymoquinone, nanothymoquinone was more effective than the parent drug in inhibiting the proliferation of human breast adenocarcinoma MCF-7 cells in a concentration-dependent manner [262]. Ultrasonic nano-emulsion formulation of the essential oil of *Nigella sativa* also exhibits anti-cancer activity as it induces apoptosis in MCF-7 cells [263]. Other studies have reported that biogenic platinum nanoparticles made using black cumin seed (*Nigella sativa L.*) extract were cytotoxic to MDA-MB-231 breast cells [264] and silver nanoparticles made from an aqueous seed extract of *Nigella sativa* induce apoptosis in MCF-7 cells by altering the expression of apoptotic proteins such as Bcl-2 and Bax and an inflammatory marker such as COX-2 [265].

The combination of thymoquinone with conventional anti-cancer drugs may improve its chemotherapeutic potential. Ganji-Harsini et al. investigated the combined effect of thymoquinone and tamoxifen on the viability of estrogen negative MDA-MB-231 human breast cancer and estrogen positive MCF-7 cells [64]. Thymoquinone and tamoxifen exerted a synergistic effect as they both decreased cell viability and induced apoptosis in both cell lines [64] (Table 2). Doxorubicin is another chemotherapy drug whose anti-tumor activity is enhanced by its combination with thymoquinone in cancer cell lines [64,251]. In a study by Arafa et al. thymoquinone induced apoptosis in doxorubicin-resistant human breast cancer MCF-7/DOX cells by the upregulation of PTEN which inhibited Akt phosphorylation [65]. There are subsequent increased cellular levels of p21 and p53 proteins, induction of G2/M cell cycle arrest and ultimately programmed cancer cell death [65] (Table 2). In another study, thymoquinone improved the anti-cancer properties of doxorubicin in MCF-7 breast cancer cell line as there was a significant increase in the inhibition of cancer cells and apoptosis. There were overall increases in selectivity, efficacy and decreased drug resistance with the use of equimolar amounts of thymoquinone and doxorubicin [266]. Moreover, seed extracts of *Nigella sativa* enhanced the antitumor activity of doxorubicin and the addition of the latter to lipid nano-emulsions of *Nigella sativa* beneficially impact the bioactivity of both drugs [267].

Paclitaxel is a chemotherapy drug used to treat patients with triple-negative breast cancer [268]. Şakalar et al. examined antitumor activity of thymoquinone and paclitaxel in a triple-negative breast cancer cell line and also in a mouse model [269]. The combination of thymoquinone and paclitaxel inhibited cancer growth in the cell culture and induced apoptosis with the upregulation of tumor suppressor genes such as Brca1, p21 and Hic1. The mechanism of action of the combined drugs also involves the downregulation of pro-apoptotic factors such as caspase-12 and caspase-7, resulting in decreased Akt and phosphorylated p65 [269]. In a recent study that examined the chemo-modulatory effect of thymoquinone on paclitaxel using MCF-7 and T47D breast cancer cells, there was decreased resistance to paclitaxel when both drugs were used in combination and increased percentage of apoptotic cell death particularly in using MCF-7 [66]. Furthermore, both drugs induced autophagy in MCF-7, and reduced breast cancer-associated stem cell clone (CD44+/CD24-cell) in both T47D and MCF-7 cancer cells [66].

Cyclophosphamide is a chemotherapy drug used to treat advanced breast cancer [270]. Khan et al. examined the synergistic effect of thymoquinone alone and in combination with cyclophosphamide on the growth of Her2- MDA-231 and Her2+ SKBR-3 breast cancer lines. The combination of thymoquinone and cyclophosphamide significantly inhibited the proliferation of cancer cells in the G1 phase, upregulated PTEN and downregulated the phosphorylation of Akt [67]. Interestingly, thymoquinone and ferulic acid from *Ferula asafetida* in combination significantly decreased the proliferation of MDA-MB-231 cancer cells [271]. A combination of thymoquinone and piperine from black pepper (*Piper longum*) acts synergistically to reduce tumor size, inhibit angiogenesis and induced apoptosis in mouse epithelial breast cancer cell line (EMT6/P) [272].

### 10.4. Clinical Studies Using Thymoquinone and Nigella sativa

There are very few clinical studies that have investigated the use of thymoquinone on breast cancer in humans. In an Arabian Phase I trial, thymoquinone administered orally at 10 mg/kg/day was well tolerated and no toxicity was reported. No side effects nor anti-cancer effects were observed at this dosage [273]. In a randomized, double-blind, placebo-controlled clinical trial comprising 62 breast cancer patients undergoing radiotherapy, *Nigella sativa* 5% gel significantly reduced the severity of acute radiation dermatitis and delays the onset of moist desquamation [97] (Table 3). The pharmacokinetic characteristics of thymoquinone such as high lipophilicity, poor solubility and low bioavailability limit its use in humans [274].

In summary, thymoquinone, the active principle from the seeds oil of *Nigella sativa* possess chemo-preventative and chemotherapeutic anti-cancer properties. The studies documented that thymoquinone inhibited the growth of breast cancer cells in animal models and culture tumors through numerous molecular mechanisms. Its delivery and efficacy as an anti-cancer agent may be improved when a very low dosage is encapsulated in nanoparticles or lipophilic biogels or when used in combination with conventional chemotherapy drugs. Thus, it is appropriate that the investigation of thymoquinone, a promising chemotherapeutic agent for the treatment of breast cancer, be moved from laboratory in vitro and in vivo experiments to clinical trials.

## 11. Conclusions

This review documented in detail the chemo-preventative and chemo-therapeutic properties of these nine herbs against breast cancer. The evidence demonstrated that the in vitro and in vivo anti-cancer effects of these herbs and their outcomes and mechanism of action include inhibition of cell proliferation, tumor growth, metastasis, angiogenesis apoptosis and regulation of cell survival pathways. The active constituents of the herbs modulate innumerable molecular events that comprise regulators of intracellular signaling such as vascular endothelial growth factor, nuclear factor-κB, and Bcl-2 that are integrally involved in the development and progression of breast cancer. However, the therapeutic efficacy of some of the biologically active compounds of herbs such as curcumin and thymoquinone are limited by their poor bioavailability and pharmacokinetic profiles, and in the case of Echinacea, the inhibition of cytochrome P450 enzymes both in vitro and in humans. These limitations can be overcome with the application of nanotechnology-based formulations and liposome carriers that indicate a feasible option for oral administration.

The anti-cancer effects of the active compounds of some of these herbs can be synergistically improved by their combination with conventional chemotherapeutic drugs such as tamoxifen, doxorubicin, 5-fluorouracil and paclitaxel. Moreover, the effectiveness of the chemotherapeutic drugs improved and there was decreased toxicity. Of interest is the synergistic effects in the co-delivery of chemotherapeutic drugs and nano-formulation of curcumin in order to improve the chemo-preventive and chemotherapeutic effects of the latter. Further studies involving large clinical trials are warranted to clarify the risk–benefit profile of the co-administration of the nano-formulation of the active compound and conventional chemotherapeutic drugs.

The majority of the chemo-preventative effects of these herbs involve a wide variety of human cancer cell lines, and to a lesser extent, animal tumor models. Therefore, while the data indicate broad anti-cancer effects of these herbs as interventions, caution should be exercised in interpretation until they can be substantiated by evidence from clinical studies. Finally, further studies of existing and novel biologically active compounds of these herbs should include quality control, toxicity and safety profiles, as well as the determination of their pharmacodynamics and pharmacokinetics. More clinical research involving trials and cohort human studies should be conducted to provide key evidence of the medical benefits of these herbs.

## Figures and Tables

**Table 1:** Anti-cancer activities of commonly studied herbs.

Herbs	Main Active Chemical Constituents	Animal Model/Tumor Cell Line	Anti-Cancer Activities/Outcome	Molecular Mechanisms/Outcome	References
Ginseng	Ginsenoside Rh2	MDA-MB-231 and MCF-7 breast cancer cell lines	Anti-proliferative and apoptosis	(i) Induce changes in hypo-methylated genes (ii) Mediate G(0)/G(1) phase cell cycle arrest (iii) inhibit the production of inflammatory cytokines (iv) Obstruct nuclear factor (NF)-κB signaling and mitogen-activated protein kinase pathways	[30,32,34,35]
Ginseng	Ginsenoside Rg3	MDA-MB-231 and MCF-7 breast cancer cell lines	Anti-proliferative	(i) Decrease expression of cyclin D1 and cyclin A (ii) Arrest cells in the G-1 phase	[36]
Garlic	Diallyl disulfide	MDA-MB-468 cancer cell line and female Swiss albino mice with EAC tumor	Decrease tumor growth and apoptosis	(i) Induce apoptosis by promoting caspase-3 expression (ii) Prevent oxidative degradation of anti-tumor protein, p53	[37]
Curcuma longa	Curcumin	MDA-MB-231 and BT-483 breast cancer cells	Anti-proliferative effect in a dose-dependent manner	(i) Downregulation of NFkappaB inducing genes (ii) Decrease transcription of matrix metalloproteinases (MMPs)-1 and cyclin D	[38]
Curcuma longa	Curcumin	MCF-7 and MDA-MB-231 breast cancer cells	Inhibition of cell proliferation and induction of apoptosis	Down-regulation of the beta-catenin pathway	[39]
Echinacea	Extracts of Echinacea purpurea	BT-549 mammalian breast cancer cell	Inhibition of cell proliferation	Mechanism not given	[40]
*Arctium lappa* (greater burdock)	Arctigenin	MDA-MB-231 breast cancer cells	Induce apoptosis	(i) Activation of the ROS/p38 MAPK pathway (ii) Induction of mitochondrial caspase-independent pathways with increased Bax/Bcl-2 ratio	[41]
Arctium lappa (greater burdock)	Arctigenin	MCF-7 and MDA-MB-231 human breast cancer cell lines	Anti-metastatic effect	Inhibiting the NF-κB, Akt/MAPK signaling pathways, and MMP-9	[42]
Flaxseed (dietary)	Lignans	Athymic mice inoculated with human MCF-7 cancer cells	Inhibition of cell proliferation and induced apoptosis	Reduced mRNA expressions of cyclin D1, epidermal growth factor receptor and Bcl2	[43]
Nigella sativa	Thymoquinone	T-47D and MDA-MB-468 breast cancer cells	Induced apoptosis	(i) Promote G (1) phase arrest via translation upregulation of procaspase-3 and Bax (ii) Inhibition of cyclin D1 and cyclin E, and PARP cleavage alongside downregulation of the gene expression of survivin, Bcl-2 and Bcl-xL	[44]
Nigella sativa	Thymoquinone	MCF-7 breast cancer cell line	Induced apoptosis	Upregulation of the expression of tumor suppressor gene p53 in a time-dependent manner	[45]
Nigella sativa	Thymoquinone	MDA-MB-231 triple-negative breast cancer cells	Anti-metastatic effect	Downregulate the expressions of CXCR4 in breast cancer cells in a time- and dose-dependent manner	[46]

**Table 2:** Studies of herbs in combination with established chemotherapy drugs.

Herbs	Main Active Chemical Constituents	Study Design - Cell Culture, Animal Model or Clinical	Anti-Cancer Drug	Endpoint and Results	References
Ginseng	Ginsenoside Rg3	MCF-7 xenografts in nude mice	Paclitaxel	(i) Enhanced the oral bioavailability of paclitaxel (ii) Improved the anti-tumor activity of paclitaxel	[54]
Cimicifuga racemose (Black cohosh)	-	Randomized controlled trial of 136 breast cancer patients	Tamoxifen	Significant reduction in the number and severity of hot flushes	[56]
Curcuma longa	Curcumin	MCF-7 and the basal-like MDA-MB-231 cancer cell lines	Paclitaxel	Synergistic therapy with (i) Decreased breast carcinogenesis by downregulating the expressions of Rho-A, p53 and Bcl-2 (ii) Decrease toxicity	[57]
Curcuma longa	Curcumin	MCF-7, SKBR3 and MDA-MB-231 breast cancer cell lines	5-fluorouracil	Increased sensitization via reducing the expression of thymidylate synthase and downregulating nuclear factor-κB	[58]
Camellia sinensis (Green tea)	Epigallocatechin gallate (EGCG) and quercetin	MCF-7 and MDA-MB-23 breast cancer cells	Tamoxifen	Synergistic activity with reduced tumor cell proliferation	[59]
Echinacea	Hexane fractions of Echinacea purpurea containing cynarin	MCF-7 breast cancer cell lines.	Doxorubicin	Enhanced cytotoxic activity of doxorubicin	[60]
Arctium lappa on	Arctigenin	(MCF7 and MDA-MB-231 breast cancer cell lines.	Doxorubicin	Synergistic effect with decreased cell viability and induced apoptosis	[61]
Flaxseed	Lignan	Athymic mice inoculated with MCF-7 breast cancer cells.	Tamoxifen	Tumor regression by over 53%	[62]
Flaxseed	Flaxseed oil (lignans)	Athymic micewith HER2-overexpressing tumor (BT-474).	Trastuzumab	Reduced phosphorylated/total expression of Akt and MAPK protein expression	[63]
Nigella sativa	Thymoquinone	MDA-MB-231 human breast cancer and estrogen positive MCF-7 cells.	Tamoxifen	Synergistic effect with decreased cell viability and induced apoptosis	[64]
Nigella sativa	Thymoquinone	MCF-7/DOX cells.	Doxorubicin	(i) Apoptosis in doxorubicin-resistant human breast cancer cells via upregulation of PTEN and inhibition of Akt phosphorylation. (ii) Increased cellular levels of p21 and p53 proteins	[65]
Nigella sativa	Thymoquinone	MCF-7 and T47D breast cancer cells.	Paclitaxel	(i) Decreased resistance to paclitacel (ii) Increased percentage of apoptotic cell death particularly in using MCF-7	[66]
Nigella sativa	Thymoquinone	Her2- MDA-231 and Her2+ SKBR-3 breast cancer lines.	Cyclophosphamide	(i) Inhibited the proliferation of cancer cells in the G1 phase (ii) Upregulated PTEN and downregulated the phosphorylation of Akt	[67]

**Table 3:** Clinical studies of the anti-cancer effects of commonly studied herbs.

Herbs	Main Active Chemical Constituents /Quantity	Study Design	Endpoints and Results	References
Garlic	-	Population-based, case control, 314 cases and 346 controls	Inverse association between breast cancer and moderate as well as high consumption	[89]
Camellia sinensis (Green tea)	Epigallocatechin-3-gallate	Case-control study of 1009 female breast cancer patients and age-matched controls	Significant protection against breast cancer (OR = 0.61)	[93]
Camellia sinensis (Green tea)	Epigallocatechin-3-gallate	472 female breast cancer patients with stage I, II and stage III disease	(i) Relative risk of recurrence of 0.564 (95% CI: 0.35 - 0.91) (ii) Prior use before diagnosis was significantly associated with better prognosis of stage I and II	[94]
Flaxseed (dietary)	Lignans	Ontario Women’s Diet and Health Study of 2,999 cases and 3,370 controls	Significant decrease in breast cancer risk (OR = 0.77)	[95]
Flaxseed (dietary)	Lignans	Randomized double-blind placebo-controlled clinical trial of postmenopausal women newly diagnosed with breast cancer	Reduced tumor growth associated with downregulation of c-erbB2 expression and reduced Ki-67 labeling index	[96]
Nigella sativa	Nigella sativa 5% gel	Randomized, double-blind, placebo-controlled clinical trial comprising 62 breast cancer patients undergoing radiotherapy	Significantly reduced the severity of acute radiation dermatitis and delays the onset of moist desquamation	[97]

## References

[1] Lundqvist A., Andersson E., Ahlberg I., Nilbert M., Gerdtham U. (2016). Socioeconomic inequalities in breast cancer incidence and mortality in Europe-a systematic review and meta-analysis. Eur. J. Public Health.

[2] Azamjah N., Soltan-Zadeh Y., Zayeri F. (2019). Global trend of breast cancer mortality rate: A 25-year study. Asian Pac. J. Cancer Prev..

[3] Hashmi A.A., Hashmi K.A., Irfan M., Khan S.M., Edhi M.M., Ali J.P., Hashmi S.K., Asif H., Faridi N., Khan A. (2019). Ki67 index in intrinsic breast cancer subtypes and its association with prognostic parameters. BMC Res. Notes.

[4] Ribnikar D., Ribeiro J.M., Pinto D., Sousa B., Pinto A.C., Gomes E., Moser E.C., Cardoso M.J., Cardoso F. (2015). Breast cancer under age 40: A different approach. Curr. Treat. Options Oncol..

[5] Maughan K.L., Lutterbie M.A., Ham P.S. (2010). Treatment of breast cancer. Am. Fam. Physician..

[6] De la Mare J.A., Contu L., Hunter M.C., Moyo B., Sterrenberg J.N., Dhanani K.C.H., Mutsvunguma L.Z., Edkins L.A. (2014). Breast cancer: Current developments in molecular approaches to diagnosis and treatment. Recent Pat. Anticancer Drug Discov..

[7] Peart O. (2015). Breast intervention and breast cancer treatment options. Radiol. Technol..

[8] Prat A., Pineda E., Adamo B., Galván P., Fernández A., Gaba L., Díez M., Viladot M., Arance A., Muñoz M. (2015). Clinical implications of the intrinsic molecular subtypes of breast cancer. Breast.

[9] Wörmann B. (2017). Breast cancer: Basics, screening, diagnostics and treatment. Grundlagen, Früherkennung, Diagnostik und Therapie. Med. Monatsschr. Pharm..

[10] Bonofiglio D., Giordano C., De Amicis F., Lanzino M., Andò S. (2016). Natural products as promising anti-tumoral agents in breast cancer: Mechanisms of action and molecular targets. Mini Rev. Med. Chem..

[11] Kumar A., Jaitak V. (2019). Natural products as multidrug resistance modulators in cancer. Eur. J. Med. Chem..

[12] Nguyen C., Baskaran K., Pupulin A., Ruvinov I., Zaitoon O., Grewal S., Scaria B., Mehaidli A., Vegh C., Pandey S. (2019). Hibiscus flower extract selectively induces apoptosis in breast cancer cells and positively interacts with common chemotherapeutics. BMC Complement. Altern. Med..

[13] Baraya Y.S., Wong K.K., Yaacob N.S. (2017). The Immunomodulatory potential of selected bioactive plant-based compounds in breast cancer: A review. Anticancer Agents Med. Chem..

[14] Křížová L., Dadáková K., Kašparovská J., Kašparovský T. (2019). Isoflavones. Molecules.

[15] Dietz B.M., Hajirahimkhan A., Dunlap T.L., Bolton J.L. (2016). Botanicals and their bioactive phytochemicals for women’s health. Pharmacol. Rev..

[16] Bak M.J., Das Gupta S., Wahler J., Suh N. (2016). Role of dietary bioactive natural products in estrogen receptor-positive breast cancer. Semin. Cancer Biol..

[17] Popovich D.G., Yeo C.R., Zhang W. (2012). Ginsenosides derived from Asian (*Panax ginseng*), American ginseng (*Panax quinquefolius*) and potential cytoactivity. Int. J. Biomed. Pharm. Sci..

[18] Yang L., Yu Q.T., Ge Y.Z., Zhang W.S., Fan Y., Ma C.W., Liu Q., Qi L.W. (2018). Distinct urine metabolome after Asian ginseng and American ginseng intervention based on GC-MS metabolomics approach. Sci. Rep..

[19] Lü J., Yao Q., Chen C. (2009). Ginseng compounds: An update on their molecular mechanisms and medical applications. Curr. Vasc. Pharmacol..

[20] Chang Y.S., Seo E., Gyllenhaal C., Block K.I. (2003). Panax ginseng: A role in cancer therapy?. Integr. Cancer Ther..

[21] Park J.D. (1996). Recent studies on the chemical constituents of Korean Ginseng (Panaxginseng C.A. Meyer). Korea J. Ginseng Sci..

[22] Yokozawa T., Kobayashi T., Oura H., Kawashima Y. (1985). Studies on the mechanism of the hypoglycemic activity of ginsenoside-Rb2 in streptozotocin-diabetic rats. Chem. Pharm. Bull..

[23] Xue J.F., Liu Z.J., Hu J.F., Chen H., Zhang J.T., Chen N.H. (2006). Ginsenoside Rb1 promotes neurotransmitter release by modulating phosphorylation of synapsins through a cAMP-dependent protein kinase pathway. Brain Res..

[24] Chen X.C., Zhou Y.C., Chen Y., Zhu Y.G., Fang F., Chen L.M. (2005). Ginsenoside Rg1 reduces MPTP induced substantia nigra neuron loss by suppressing oxidative stress. Acta Pharmacol. Sin..

[25] Harkey M.R., Henderson G.L., Gershwin M.E., Stern J.S., Hackman R.M. (2001). Variability in commercial ginseng products: An analysis of 25 preparations. Am. J. Clin. Nutr..

[26] Mizuno M., Yamada J., Terai H., Kozukue N., Lee Y.S., Tsuchida H. (1994). Differences in immune-modulating effects between wild and cultured Panax ginseng. Biochem. Biophys. Res. Commun..

[27] Nag S.A., Nag J.J., Qin W., Wang M.H., Wang H., Zhang R. (2012). Ginsenosides as anticancer agents: In vitro and in vivo activities, structure-activity relationships, and molecular mechanisms of action. Front. Pharmacol..

[28] Riaz M., Rahman N., Zia-Ul-Haq M., Jaffar H., Manea R. (2019). Ginseng: A dietary supplement as immune-modulator in various diseases. Trends Food Sci. Technol..

[29] Chen X.J., Zhang X.J., Shui Y.M., Wan J.B., Ga J.L. (2016). Anticancer activities of Protopanaxadiol- and Protopanaxatriol-Type Ginsenosides and their metabolites. Evid. Based Complement. Altern. Med..

[30] Lee H., Lee S., Jeong D., Kim S.J. (2018). Ginsenoside Rh2 epigenetically regulates cell-mediated immune pathway to inhibit proliferation of MCF-7 breast cancer cells. J. Ginseng Res..

[31] Jeong D., Ham J., Park S., Kim H.W., Kim H., Ji H.W., Kim S.J. (2018). Ginsenoside Rh2 suppresses breast cancer cell proliferation by epigenetically regulating the long noncoding RNA C3orf67-AS1. J. Ginseng Res..

[32] Choi S., Kim T.W., Singh S.V. (2009). Ginsenoside Rh2-mediated G1 phase cell cycle arrest in human breast cancer cells is caused by p15 Ink4B and p27 Kip1-dependent inhibition of cyclin-dependent kinases. Pharm. Res..

[33] Oh M., Choi Y.H., Choi S., Chung H., Kim K., Kim D.K., Kim N.D. (1999). Anti-proliferating effects of ginsenoside Rh2 on MCF-7 human breast cancer cells. Int. J. Oncol..

[34] Bi W.Y., Fu B.D., Shen H.Q., Wei Q., Zhang C., Song Z., Qin Q.Q., Li H.P., Lv S., Wu S.C. (2012). Sulfated derivative of 20(S)-ginsenoside Rh2 inhibits inflammatory cytokines through MAPKs and NF-kappa B pathways in LPS-induced RAW264.7 macrophages. Inflammation.

[35] Zhang J., Lu M., Zhou F., Sun H., Hao G., Wu X., Wang G. (2012). Key role of nuclear factor-kappaB in the cellular pharmacokinetics of adriamycin in MCF-7/Adr cells: The potential mechanism for synergy with 20(S)-ginsenoside Rh2. Drug Metab. Dispos..

[36] Wang C.-Z., Aung H.H., Zhang B., Sun S., Li X.-L., He H., Xie J.-T., He T.-C., Du W., Yuan C.-S. (2008). Chemo-preventive effects of heat-processed Panax quinquefolius root on human breast cancer cells. Anticancer Res..

[37] Sujatha P., Anantharaju P.G., Veeresh P.M., Dey S., Bovilla V.R., Madhunapantula S.R.V. (2017). Diallyl disulfide (DADS) retards the growth of breast cancer cells in vitro and in vivo through apoptosis induction. Biomed. Pharmacol. J..

[38] Liu Q., Loo W.T., Sze S.C., Tong Y. (2009). Curcumin inhibits cell proliferation of MDA-MB-231 and BT-483 breast cancer cells mediated by down-regulation of NFkappaB, cyclin D and MMP-1 transcription. Phytomedicine.

[39] Prasad C.P., Rath G., Mathur S., Bhatnagar D., Ralhan R. (2009). Potent growth suppressive activity of curcumin in human breast cancer cells: Modulation of Wnt/β-catenin signaling. Chem. Biol. Interact..

[40] Driggins S.N., Myles E.L., Gary T. (2004). The anti-prolific effect of *Echinacea Pallida* on BT-549 cancer cell line. Cancer Res..

[41] Hsieh C., Kuo P., Hsu Y., Huang Y., Tsai E., Hsu Y. (2014). Arctigenin, a dietary phytoestrogen, induces apoptosis of estrogen receptor-negative breast cancer cells through the ROS/p38 MAPK pathway and epigenetic regulation. Free Radic. Biol. Med..

[42] Maxwell T., Chun S.Y., Lee K.S., Kim S., Nam K.S. (2017). The anti-metastatic effects of the phytoestrogen arctigenin on human breast cancer cell lines regardless of the status of ER expression. Int. J. Oncol..

[43] Chen J., Saggar J.K., Corey P., Thompson L.U. (2009). Flaxseed and pure secoisolariciresinol diglucoside, but not flaxseed hull, reduce human breast tumor growth (MCF-7) in athymic mice. J. Nutr..

[44] Rajput S., Kumar B.N., Dey K.K., Pal I., Parekh A., Mandal M. (2013). Molecular targeting of Akt by thymoquinone promotes G(1) arrest through translation inhibition of cyclin D1 and induces apoptosis in breast cancer cells. Life Sci..

[45] Dastjerdi M.N., Mehdiabady E.M., Iranpour F.G., Bahramian H. (2016). Effect of thymoquinone on P53 gene expression and consequence apoptosis in breast cancer cell line. Int. J. Prev. Med..

[46] Shanmugam M.K., Ahn K.S., Hsu A., Woo C.C., Yuan Y., Tan K.H.B., Chinnathambi A., Alahmadi T.A., Alharbi S.A., Koh A.P.F. (2018). Thymoquinone inhibits bone metastasis of breast cancer cells through abrogation of the CXCR4 signaling axis. Front Pharmacol..

[47] Ham J., Lee S., Lee H., Jeong D., Park S., Kim S.J. (2018). Genome-wide methylation analysis identifies NOX4 and KDM5A as key regulators in inhibiting breast cancer cell proliferation by Ginsenoside Rg3. Am. J. Chin. Med..

[48] Zou M., Wang J., Gao J., Han H., Fang Y. (2018). Phosphoproteomic analysis of the antitumor effects of ginsenoside Rg3 in human breast cancer cells. Oncol. Lett..

[49] Kim B.M., Kim D.H., Park J.H., Na H.K., Surh Y.J. (2013). Ginsenoside Rg3 induces apoptosis of human breast cancer (MDA-MB-231) cells. J. Cancer Prev..

[50] Kim B.M., Kim D.H., Park J.H., Surh Y.J., Na H.K. (2014). Ginsenoside Rg3 inhibits constitutive activation of NF-κB signaling in human breast cancer (MDA-MB-231) cells: ERK and Akt as potential upstream Targets. J. Cancer Prev..

[51] Tang H., Ren Y., Zhang J., Ma S., Gao F., Wu Y. (2007). Correlation of insulin-like growth factor-1 (IGF-1) to angiogenesis of breast cancer in IGF-1-deficient mice. Ai Zheng Aizheng Chin. J. Cancer.

[52] Chen X., Qian L., Jiang H., Chen J.H. (2011). Ginsenoside Rg3 inhibits CXCR_4_ expression and related migrations in a breast cancer cell line. Int. J. Clin. Oncol..

[53] Mukherjee D., Zhao J. (2013). The role of chemokine receptor CXCR4 in breast cancer metastasis. Am. J. Cancer Res..

[54] Yang L.Q., Wang B., Gan H., Fu S.T., Zhu X.X., Wu Z.N., Zhan D.W., Gu R.L., Dou G.F., Meng Z.Y. (2012). Enhanced oral bioavailability and anti-tumour effect of paclitaxel by 20 (s)-ginsenoside Rg3 in vivo. Biopharm. Drug Dispos..

[55] Zhang Q., Kang X., Yang B., Wang J., Yang F. (2008). Antiangiogenic effect of capecitabine combined with ginsenoside Rg3 on breast cancer in mice. Cancer Biother. Radiopharm..

[56] Hernández Muñoz G., Pluchino S. (2003). Cimicifuga racemosa for the treatment of hot flushes in women surviving breast cancer. Maturitas.

[57] Quispe-Soto E.T., Calaf G.M. (2016). Effect of curcumin and paclitaxel on breast carcinogenesis. Int. J. Oncol..

[58] Vinod B.S., Antony J., Nair H.H., Puliyappadamba V.T., Saikia M., Narayanan S.S., Bevin A., Anto R.J. (2013). Mechanistic evaluation of the signaling events regulating curcumin-mediated chemo-sensitization of breast cancer cells to 5-fluorouracil. Cell Death Dis..

[59] Schroeder E.K., Kelsey N.A., Doyle J., Breed E., Bouchard R.J., Loucks F.A., Harbison R.A., Linseman D.A. (2009). Green tea epigallocatechin 3-gallate accumulates in mitochondria and displays a selective anti-apoptotic effect against inducers of mitochondrial oxidative stress in neurons. Antioxid. Redox Signal..

[60] Goey A.K.L., Meijerman I., Rosing H., Burgers J.A., Mergui-Roelvink M., Keessen M., Marchetti S., Beijnen J.H., Schellens J.H.M. (2013). The effect of *Echinacea purpurea* on the pharmacokinetics of docetaxel. Br. J. Clin. Pharmacol..

[61] Ghafari F., Rajabi M.R., Mazoochi T., Taghizadeh M., Nikzad H., Atlasi M.A., Taherian A. (2017). Comparing apoptosis and necrosis effects of *Arctium Lappa* root extract and doxorubicin on MCF7 and MDA-MB-231 cell lines. Asian Pac. J. Cancer Prev..

[62] Chen J., Hui E., Ip T., Thompson L.U. (2004). Dietary flaxseed enhances the inhibitory effect of tamoxifen on the growth of estrogen-dependent human breast cancer (mcf-7) in nude mice. Clin. Cancer Res..

[63] Mason J.K., Fu M., Chen J., Thompson L.U. (2015). Flaxseed oil enhances the effectiveness of trastuzumab in reducing the growth of HER2-overexpressing human breast tumors (BT-474). J. Nutr. Biochem..

[64] Ganji-Harsini S., Khazaei M., Rashidi Z., Ghanbari A. (2016). Thymoquinone could increase the efficacy of tamoxifen induced apoptosis in human breast cancer cells: An in vitro study. Cell J..

[65] Arafa E.A., Zhu Q., Shah Z.I., Wani G., Barakat B.M., Racoma I., El-Mahdy M.A., Wani A.A. (2011). Thymoquinone up-regulates PTEN expression and induces apoptosis in doxorubicin-resistant human breast cancer cells. Mutat. Res..

[66] Bashmail H.A., Alamoudi A.A., Noorwali A., Hegazy G.A., Ajabnoor G.M., Al-Abd A.M. (2020). Thymoquinone enhances paclitaxel anti-breast cancer activity via inhibiting tumor-associated stem cells despite apparent mathematical antagonism. Molecules.

[67] Khan A., Aldebasi Y.H., Alsuhaibani S.A., Khan M.A. (2019). Thymoquinone augments cyclophosphamide-mediated inhibition of cell proliferation in breast cancer cells. Asian Pac. J. Cancer Prev..

[68] Chen S., Wang Z., Huang Y., O’Barr S.A., Wong R.A., Yeung S., Chow M.S. (2014). Ginseng and anticancer drug combination to improve cancer chemotherapy: A critical review. Evid. Based Complement. Altern. Med..

[69] Cui Y., Shu X.O., Gao Y.T., Cai H., Tao M.H., Zheng W. (2006). Association of ginseng use with survival and quality of life among breast cancer patients. Am. J. Epidemiol..

[70] Bao P.P., Lu W., Cui Y., Zheng Y., Gu K., Chen Z., Zheng W., Shu X.O. (2012). Ginseng and Ganoderma lucidum use after breast cancer diagnosis and quality of life: A report from the Shanghai Breast Cancer Survival Study. PLoS ONE.

[71] Block E. (2010). Garlic and Other Alliums: The Lore and the Science.

[72] Amagase H. (2006). Clarifying the real bioactive constituents of garlic. J. Nutr..

[73] Kourounakis P.N., Rekka E.A. (1991). Effect on active oxygen species of alliin and *Allium sativum* (garlic) powder. Res. Commun. Chem. Pathol. Pharmacol..

[74] Gorinstein S., Drzewiecki J., Leontowicz H., Leontowicz M., Najman K., Jastrzebski Z., Zachwieja Z., Barton H., Shtabsky B., Katrich E. (2005). Comparison of the bioactive compounds and antioxidant potentials of fresh and cooked Polish, Ukrainian, and Israeli garlic. J. Agric. Food Chem..

[75] Thomson M., Ali M. (2003). Garlic allium sativum: A review of its potential use as an anti-cancer agent. Curr. Cancer Drug Targets.

[76] Dahanukar S.A., Thatte U.M. (1997). Current status of ayurveda in phytomedicine. Phytomedicine.

[77] Milner J.A. (1996). Garlic: Its anti-carcinogenic and anti-tumorigenic properties. Nutr. Rev..

[78] Knowles L.M., Milner J.A. (2001). Possible mechanism by which allyl sulfides suppress neoplastic cell proliferation. J. Nutr..

[79] Bayan L., Koulivand P.H., Gorji A. (2014). Garlic: A review of potential therapeutic effects. Avicenna J. Phytomed..

[80] Omar S.H., Al-Wabel N.A. (2010). Organosulfur compounds and possible mechanism of garlic in cancer. Saudi Pharm. J..

[81] Tsubura A., Lai Y.C., Kuwata M., Uehara N., Yoshizawa K. (2011). Anticancer effects of garlic and garlic-derived compounds for breast cancer control. Anticancer Agents Med. Chem..

[82] Modem S., Dicarlo S.E., Reddy T.R. (2012). Fresh garlic extract induces growth arrest and morphological differentiation of MCF7 breast cancer cells. Genes Cancer.

[83] Ghazanfari T., Yaraee R., Rahmati B., Hakimzadeh H., Shams J., Jalali-Nadoushan M.R. (2011). In vitro cytotoxic effect of garlic extract on malignant and nonmalignant cell lines. Immunopharmacol. Immunotoxicol..

[84] Bagul M., Kakumanu S., Wilson T.A. (2015). Crude garlic extract inhibits cell proliferation and induces cell cycle arrest and apoptosis of cancer cells in vitro. J. Med. Food.

[85] Hirsch K., Danilenko M., Giat J., Miron T., Rabinkov A., Wilchek M., Mirelman J., Levy J., Sharoni Y. (2000). Effect of purified allicin, the major ingredient of freshly crushed garlic, on cancer cell proliferation. Nutr Cancer.

[86] Malki A., El-Saadani M., Sultan A.S. (2009). Garlic constituent diallyl trisulfide induced apoptosis in MCF7 human breast cancer cells. Cancer Biol. Ther..

[87] Fleischauer A.T., Arab L. (2001). Garlic and cancer: A critical review of the epidemiologic literature. J. Nutr..

[88] Alizadeh-Navaei R., Shamshirian A., Hedayatizadeh-Omran A., Ghadimi R., Janbabai G. (2018). Effect of garlic in gastric cancer prognosis: A systematic review and meta-analysis. World Cancer Res. J..

[89] Desai G., Schelske-Santos M., Nazario C.M., Rosario-Rosado R.V., Mansilla-Rivera I., Ramírez-Marrero F., Nie J., Myneni A.A., Zhang Z.F., Freudenheim J.L. (2019). Onion and garlic intake and breast cancer, a case-control study in Puerto Rico. Nutr. Cancer..

[90] Challier B., Perarnau J.M., Viel J.F. (1998). Garlic, onion and cereal fibre as protective factors for breast cancer: A French case-control study. Eur. J. Epidemiol..

[91] Galeone C., Pelucchi C., Levi F., Negri E., Franceschi S., Talamini R., Giacosa A., La Vecchia C. (2006). Onion and garlic use and human cancer. Am. J. Clin. Nutr..

[92] Kim J.Y., Kwon O. (2009). Garlic intake and cancer risk: An analysis using the Food and Drug Administration’s evidence-based review system for the scientific evaluation of health claims. Am. J. Clin. Nutr..

[93] Zhang M., Holman C.D., Huang J.P., Xie X. (2007). Green tea and the prevention of breast cancer: A case-control study in Southeast China. Carcinogenesis.

[94] Nakachi K., Suemasu K., Suga K., Takeo T., Imai K., Higashi Y. (1998). Influence of drinking green tea on breast cancer malignancy among Japanese patients. Jpn. J. Cancer Res. Gann.

[95] Lowcock E.C., Cotterchio M., Boucher B.A. (2013). Consumption of flaxseed, a rich source of lignans, is associated with reduced breast cancer risk. Cancer Causes Control.

[96] Thompson L.U., Chen J.M., Li T., Strasser-Weippl K., Goss P.E. (2005). Dietary flaxseeds alters tumor biological markers in postmenopausal breast cancer. Clin. Cancer Res..

[97] Rafati M., Ghasemi A., Saeedi M., Habibi E., Salehifar E., Mosazadeh M., Maham M. (2019). *Nigella sativa* L. for prevention of acute radiation dermatitis in breast cancer: A randomized, double-blind, placebo-controlled, clinical trial. Complement. Ther. Med..

[98] Predny M.L., De Angelis P., Chamberlain J.L. (2006). Black Cohosh (Actaea racemosa): An Annotated Bibliography.

[99] Chen S.N., Li W.K., Fabricant D.S., Santarsiero B.D., Mesecar A., Fitzloff J.F., Fong H.S., Farnsworth N.R. (2002). Isolation, structure elucidation, and absolute configuration of 26-deoxyactein from Cimicifuga racemosa and clarification of nomenclature associated with 27-deoxyactein. J. Nat. Prod..

[100] Shao Y., Harris A., Wang M., Zhang H., Cordell G.A., Bowman M., Lemmo E. (2000). Triterpene glycosides from Cimicifuga racemosa. J. Nat. Prod..

[101] Jiang B., Kronenberg F., Nuntanakorn P., Qiu M.H., Kennelly E.J. (2006). Evaluation of the botanical authenticity and phytochemical profile of black cohosh products by high-performance liquid chromatography with selected ion monitoring liquid chromatography-mass spectrometry. J. Agric. Food Chem..

[102] Chen S.N., Fabricant D.S., Lu Z.Z., Zhang H.J., Fong H.S., Farnsworth N.R. (2002). Cimiracemates A-D phenylpropanoid esters from the rhizomes of Cimicifuga racemosa. Phytochemistry.

[103] Jarry H., Harnischfeger G., Düker E.M. (1985). Studies on the endocrine efficacy of the constituents of Cimicifuga racemosa: 2. In vitro binding of constituents to estrogen receptors. Planta Med..

[104] Kligler B. (2003). Black cohosh. Am. Fam. Physician.

[105] Baber R., Hickey M., Kwik M. (2005). Therapy for menopausal symptoms during and after treatment for breast cancer: Safety considerations. Drug Saf..

[106] Carroll D.G. (2006). Non-hormonal therapies for hot flashes in menopause. Am. Fam. Physician.

[107] Vermes G., Banhidy F., Acs N. (2005). The effects of Remifemin on subjective symptoms of menopause. Adv. Ther..

[108] Geller S.E., Shulman L.P., van Breemen R.B., Banuvar S., Zhou Y., Epstein G., Hedayat S., Nikolic D., Krause E.C., Piersen C.E. (2009). Safety and efficacy of black cohosh and red clover for the management of vasomotor symptoms: A randomized controlled trial. Menopause (New York, N.Y.).

[109] Nuntanakorn P., Jiang B., Yang H., Cervantes-Cervantes M., Kronenberg F., Kennelly E.J. (2007). Analysis of polyphenolic compounds and radical scavenging activity of four American Actaea species. Phytochem. Anal..

[110] Wuttke W., Seidlová-Wuttke D. (2015). Black cohosh (*Cimicifuga racemosa*) is a non-estrogenic alternative to hormone replacement therapy. Clin. Phytosci..

[111] Hoda D., Perez D.G., Loprinzi C.L. (2003). Hot flashes in breast cancer survivors. Breast J..

[112] Ruhlen R.L., Sun G.Y., Sauter E.R. (2008). Black cohosh: Insights into its mechanism(s) of action. Integr. Med. Insights.

[113] Rostock M., Fischer J., Mumm A., Stammwitz U., Saller R., Bartsch H.H. (2011). Black cohosh (*Cimicifuga racemosa*) in tamoxifen-treated breast cancer patients with climacteric complaints—A prospective observational study. Gynecol. Endocrinol..

[114] Pockaj B.A., Loprinzi C.L., Sloan J.A., Novotny P.J., Barton D.L., Hagenmaier A., Zhang H., Lambert G.H., Reeser K.A., Wisbey J.A. (2004). Pilot evaluation of black cohosh for the treatment of hot flashes in women. Cancer Investig..

[115] Pockaj B.A., Gallagher J.G., Loprinzi C.L., Stella P.J., Barton D.L., Sloan J.A., Lavasseur B.I., Rao R.M., Fitch T.R., Rowland K.M. (2006). Phase III double-blind, randomized, placebo-controlled crossover trial of black cohosh in the management of hot flashes: NCCTG Trial N01CC1. J. Clin. Oncol..

[116] Jacobson J.S., Troxel A.B., Evans J., Klaus L., Vahdat L., Kinne D., Lo K.M., Rosenman E., Kaufman A., Neugut A.I. (2001). Randomized trial of black cohosh for the treatment of hot flashes among women with a history of breast cancer. J. Clin. Oncol..

[117] Priyadarsini K.I. (2014). The chemistry of curcumin: From extraction to therapeutic agent. Molecules.

[118] Amalraj A., Pius A., Gopi S., Gopi S. (2017). Biological activities of curcuminoids, other biomolecules from turmeric and their derivatives—A review. J. Tradit. Complement. Med..

[119] Krup V., Prakash H.L., Harini A. (2013). Pharmacological activities of turmeric (Curcuma longa Linn): A review. J. Tradit. Med. Clin. Naturop..

[120] Gupta S.C., Patchva S., Aggarwal B.B. (2013). Therapeutic roles of curcumin: Lessons learned from clinical trials. AAPS J..

[121] Rai M., Pandit R., Gaikwad S., Yadav A., Gade A. (2015). Potential applications of curcumin and curcumin nanoparticles: From traditional therapeutics to modern nanomedicine. Nanotechnol. Rev..

[122] Patel S.S., Acharya A., Ray R.S., Agrawal R., Raghuwanshi R., Jain P. (2020). Cellular and molecular mechanisms of curcumin in prevention and treatment of disease. Crit. Rev. Food Sci. Nutr..

[123] Liu C., Zhu L., Fukuda K., Ouyang S., Chen X., Wang C., Zhang C.J., Martin B., Gu C., Qin L. (2017). The flavonoid cyanidin blocks binding of the cytokine interleukin-17A to the IL-17RA subunit to alleviate inflammation in vivo. Sci. Signal..

[124] Zong H., Wang F., Fan Q.X., Wang L.X. (2012). Curcumin inhibits metastatic progression of breast cancer cell through suppression of urokinase-type plasminogen activator by NF-kappa B signaling pathways. Mol. Biol. Rep..

[125] Van den Eijnden M.J., Strous J.G. (2007). Autocrine growth hormone: Effects on growth hormone receptor trafficking and signaling. Mol. Endocrinol..

[126] Coker-Gurkan A., Bulut D., Genc R., Arisan E.D., Obakan-Yerlikaya P., Palavan-Unsal N. (2019). Curcumin prevented human autocrine growth hormone (GH) signaling mediated NF-κB activation and miR-183-96-182 cluster stimulated epithelial mesenchymal transition in T47D breast cancer cells. Mol. Biol. Rep..

[127] Coker-Gurkan A., Celik M., Ugur M., Arisan E.D., Obakan-Yerlikaya P., Durdu Z.B., Palavan-Unsal N. (2018). Curcumin inhibits autocrine growth hormone-mediated invasion and metastasis by targeting NF-κB signaling and polyamine metabolism in breast cancer cells. Amino Acids.

[128] Porta C., Paglino C., Mosca A. (2014). Targeting PI3K/Akt/mTOR signaling in cancer. Front. Oncol..

[129] Guan F., Ding Y., Zhang Y., Zhou Y., Li M., Wang C. (2016). Curcumin suppresses proliferation and migration of MDA-MB-231 breast cancer cells through autophagy-dependent Akt degradation. PLoS ONE.

[130] Papaliagkas V., Anogianaki A., Anogianakis G., Ilonidis G. (2007). The proteins and the mechanisms of apoptosis: A mini-review of the fundamentals. Hippokratia.

[131] Akkoç Y., Berrak Ö., Arısan E.D., Obakan P., Çoker-Gürkan A., Palavan-Ünsal N. (2015). Inhibition of PI3K signaling triggered apoptotic potential of curcumin which is hindered by Bcl2 through activation of autophagy in MCF-7 cells. Biomed. Pharmacother..

[132] Lai H.W., Chien S.Y., Kuo S.J., Tseng L.M., Lin H.Y., Chi C.W., Chen D.R. (2012). The potential utility of curcumin in the treatment of HER-2-Overexpressed breast cancer: An in vitro and in vivo comparison study with Herceptin. Evid. Based Complement. Altern. Med..

[133] Loh H.Y., Norman B.P., Lai K.S., Rahman N., Alitheen N., Osman M.A. (2019). The regulatory role of MicroRNAs in breast cancer. Int. J. Mol. Sci..

[134] Wang X., Hang Y., Liu J., Hou Y., Wang N., Wang M. (2017). Anticancer effect of curcumin inhibits cell growth through miR-21/PTEN/Akt pathway in breast cancer cell. Oncol. Lett..

[135] Li X., Xie W., Xie C., Huang C., Zhu J., Liang Z., Deng F., Zhu M., Zhu W., Wu R. (2014). Curcumin modulates miR-19/PTEN/AKT/p53 axis to suppress bisphenol A-induced MCF-7 breast cancer cell proliferation. Phytother. Res..

[136] Yang J., Cao Y., Sun J., Zhang Y. (2010). Curcumin reduces the expression of Bcl-2 by upregulating miR-15a and miR-16 in MCF-7 cells. Med. Oncol..

[137] Norouzi S., Majeed M., Pirro M., Generali D., Sahebkar A. (2018). Curcumin as an adjunct therapy and microRNA modulator in breast cancer. Curr. Pharm. Des..

[138] Zhan Y., Chen Y., Liu R., Zhang H., Zhang Y. (2014). Potentiation of paclitaxel activity by curcumin in human breast cancer cell by modulating apoptosis and inhibiting EGFR signaling. Arch. Pharm. Res..

[139] Bayet-Robert M., Morvan D. (2013). Metabolomics reveals metabolic targets and biphasic responses in breast cancer cells treated by curcumin alone and in association with docetaxel. PLoS ONE.

[140] Ponce-Cusi R., Ponce-Cusi R. (2016). Apoptotic activity of 5-fluorouracil in breast cancer cells transformed by low doses of ionizing α-particle radiation. Int. J. Oncol..

[141] Zou J., Zhu L., Jiang X., Wang Y., Wang Y., Wang X., Chen B. (2018). Curcumin increases breast cancer cell sensitivity to cisplatin by decreasing FEN1 expression. Oncotarget.

[142] Wen C., Fu L., Huang J., Dai Y., Wang B., Xu G., Wu L., Zhou H. (2019). Curcumin reverses doxorubicin resistance via inhibition the efflux function of ABCB4 in doxorubicin-resistant breast cancer cells. Mol. Med. Rep..

[143] Jiang M., Huang O., Zhang X., Xie Z., Shen A., Liu H., Geng M., Shen K. (2013). Curcumin induces cell death and restores tamoxifen sensitivity in the antiestrogen-resistant breast cancer cell lines MCF-7/LCC2 and MCF-7/LCC9. Molecules.

[144] Mock C.D., Jordan B.C., Selvam C. (2015). Recent advances of curcumin and its analogues in breast cancer prevention and treatment. RSC Adv..

[145] Nejati-Koshki K., Akbarzadeh A., Pourhasan-Moghaddam M., Abhari A., Dariushnejad H. (2014). Inhibition of leptin and leptin receptor gene expression by silibinin-curcumin combination. Asian Pac. J. Cancer Prev..

[146] Graham H.N. (1992). Green tea composition, consumption, and polyphenol chemistry. Prev. Med..

[147] Corcoran M.P., McKay D.L., Blumberg J.B. (2012). Flavonoid basics: Chemistry, sources, mechanisms of action, and safety. J. Nutr. Gerontol. Geriatr..

[148] Cabrera C., Artacho R., Gimenez R. (2006). Beneficial effects of green tea: A review. J. Am. Coll. Nutr..

[149] Sano M., Tabata M., Suzuki M., Degawa M., Miyase T., Maeda-Yamamoto M. (2001). Simultaneous determination of twelve tea catechins by high-performance liquid chromatography with electrochemical detection. Analyst.

[150] Fernandez P.L., Martin M.J., Gonzalez A.G., Pablos F. (2000). HPLC determination of catechins and caffeine in tea. Differentiation of green, black and instant teas. Analyst.

[151] Chacko S.M., Thambi P.T., Kuttan R., Nishigaki I. (2010). Beneficial effects of green tea: A literature review. Chin. Med..

[152] Koo M.W., Cho C.H. (2004). Pharmacological effects of green tea on the gastrointestinal system. Eur. J. Pharmacol..

[153] Zaveri N.T. (2006). Green tea and its polyphenolic catechins: Medicinal uses in cancer and non-cancer applications. Life Sci..

[154] Legeay S., Rodier M., Fillon L., Faure S., Clere N. (2015). Epigallocatechin gallate: A review of its beneficial properties to prevent metabolic syndrome. Nutrients.

[155] Zhang D., Nichols H.B., Troester M., Cai J., Bensen J.T., Sandler D.P. (2019). Tea consumption and breast cancer risk in a cohort of women with family history of breast cancer. Int. J. Cancer.

[156] Zhang J.Y., Liao Y.H., Lin Y., Liu Q., Xie X.M., Tang L.Y., Ren Z.F. (2019). Effects of tea consumption and the interactions with lipids on breast cancer survival. Breast Cancer Res. Treat..

[157] Li M., Tse L.A., Chan W.C., Kwok C.H., Leung S.L., Wu C., Yu W.C., Yu I.T., Yu C.H., Wang F. (2016). Evaluation of breast cancer risk associated with tea consumption by menopausal and estrogen receptor status among Chinese women in Hong Kong. Cancer Epidemiol..

[158] Boggs D.A., Palmer J.R., Stampfer M.J., Spiegelman D., Adams-Campbell L.L., Rosenberg L. (2010). Tea and coffee intake in relation to risk of breast cancer in the Black Women’s Health Study. Cancer Causes Control.

[159] Wu A.H., Butler L.M. (2011). Green tea and breast cancer. Mol. Nutr. Food Res..

[160] Gianfredi V., Nucci D., Abalsamo A., Acito M., Villarini M., Moretti M., Realdon S. (2018). Green tea consumption and risk of breast cancer and recurrence—A systematic review and meta-analysis of observational studies. Nutrients.

[161] Ogunleye A.A., Xue F., Michels K.B. (2010). Green tea consumption and breast cancer risk or recurrence: A meta-analysis. Breast Cancer Res. Treat..

[162] Seely D., Mills E.J., Wu P., Verma S., Guyatt H.H. (2005). The effects of green tea consumption on incidence of breast cancer and recurrence of breast cancer: A systematic review and meta-analysis. Integr. Cancer Ther..

[163] Najafi N., Salehi M., Ghazanfarpour M., Hoseini Z.S., Khadem-Rezaiyan M. (2018). The association between green tea consumption and breast cancer risk: A systematic review and meta-analysis. Phytother. Res..

[164] Luo J., Gao Y.T., Chow W.H., Shu X.O., Li H., Yang G., Cai Q., Rothman N., Cai H., Shrubsole M.J. (2010). Urinary polyphenols and breast cancer risk: Results from the Shanghai Women’s Health Study. Breast Cancer Res. Treat..

[165] Iwasaki M., Inoue M., Sasazuki S., Miura T., Sawada N., Yamaji T., Shimazu T., Willett W.C., Tsugane S. (2010). Plasma tea polyphenol levels and subsequent risk of breast cancer among Japanese women: A nested case-control study. Breast Cancer Res. Treat..

[166] Nazari S.S., Mukherjee P. (2018). An overview of mammographic density and its association with breast cancer. Breast Cancer.

[167] Wu A.H., Ursin G., Koh W.P., Wang R., Yuan J.M., Khoo K.S., Yu M.C. (2008). Green tea, soy, and mammographic density in Singapore Chinese women. Cancer Epidemiol. Biomark. Prev..

[168] Samavat H., Ursin G., Emory T.H., Lee E., Wang R., Torkelson C.J., Dostal A.M., Swenson K., Le C.T., Yang C.S. (2017). A randomized controlled trial of green tea extract supplementation and mammographic density in postmenopausal women at increased risk of breast cancer. Cancer Prev. Res..

[169] Miller R.J., Jackson K.G., Dadd T., Nicol B., Dick J.L., Mayes A.E., Brown A.L., Minihane A.M. (2012). A preliminary investigation of the impact of catechol-O-methyltransferase genotype on the absorption and metabolism of green tea catechins. Eur. J. Nutr..

[170] Wu A.H., Tseng C.C., Van Den Berg D., Yu M.C. (2003). Tea intake, COMT genotype, and breast cancer in Asian-American women. Cancer Res..

[171] Shrubsole M.J., Lu W., Chen Z., Shu X.O., Zheng Y., Dai Q., Cai Q., Gu K., Ruan Z.X., Gao Y.-T. (2009). Drinking green tea modestly reduces breast cancer risk. J. Nutr..

[172] Tyagi T., Treas J.N., Mahalingaiah P.K., Singh K.P. (2015). Potentiation of growth inhibition and epigenetic modulation by combination of green tea polyphenol and 5-aza-2′-deoxycytidine in human breast cancer cells. Breast Cancer Res. Treat..

[173] Lewis K.A., Jordan H.R., Tollefsbol T.O. (2018). Effects of SAHA and EGCG on growth potentiation of triple-negative breast cancer cells. Cancers.

[174] Zhou Y., Tang J., Du Y., Ding J., Liu J.Y. (2016). The green tea polyphenol EGCG potentiates the antiproliferative activity of sunitinib in human cancer cells. Tumour Biol..

[175] Goldhaber-Fiebert S., Kemper K. (1999). Echinacea: E. angustifolia, E. pallida, and E. purpurea. Center Holist. Pediatr. Educ. Res..

[176] Brown P., Chan M., Paley L., Betz J.M. (2011). Determination of major phenolic compounds in Echinacea spp. Raw materials and finished products by High-Performance Liquid Chromatography with ultraviolet detection: Single laboratory validation matrix extension. J. AOAC Int..

[177] Kumar K.M., Sudha R.S. (2011). Pharmacological importance of *Echinacea purpurea*. Int. J. Pharma. Bio Sci..

[178] Manayi A., Vazirian M., Saeidnia S. (2015). *Echinacea purpurea*: Pharmacology, phytochemistry and analysis methods. Pharmacogn. Rev..

[179] Werneke U., Earl J., Seydel C., Horn O., Crichton P., Fannon D. (2004). Potential health risks of complementary alternative medicines in cancer patients. Br. J. Cancer.

[180] Ma H., Carpenter C.L., Sullivan-Halley J., Bernstein L. (2011). The roles of herbal remedies in survival and quality of life among long-term breast cancer survivors—Results of a prospective study. BMC Cancer.

[181] Bright-Gbebry M., Makambi K.H., Rohan J.P., Llanos A.A., Rosenberg L., Palmer J.R., Adams-Campbell L.L. (2011). Use of multivitamins, folic acid and herbal supplements among breast cancer survivors: The black women’s health study. BMC Complement. Altern. Med..

[182] Chica A., Adinolfi B., Pellati F., Orlandini G., Benvenuti S., Nieri P. (2010). Cytotoxic activity and G1 cell cycle arrest of a Dienynone from *Echinacea pallida*. Planta Med..

[183] Modearai M., Gertsch J., Suter A., Heinrich M., Kortenkamp A. (2007). Cytochrome P450 inhibitory action of Echinacea preparations differs widely and co-varies with alkylamide content. J. Pharm. Pharmacol..

[184] Beshay N.M.Z., Ayman O.S.E. (2008). Induction of several cytochrome P450 genes by doxorubicin in H9c2 cells. Vasc. Pharmacol..

[185] Huntimer E.D., Halaweish F.T., Chase C.C.L. (2006). Proliferative activity of *Echinacea angustifolia* root extracts on cancer cells: Interference with doxorubicin cytotoxicity. Chem. Biodivers..

[186] Coelho de Souza G., Haas A.P.S., von Poser G.L., Schapoval E.E.S., Elisabetsky E. (2004). Ethnopharmacological studies of antimicrobial remedies in the south of Brazil. J. Ethnopharmacol..

[187] Lopez-Vinyallonga S., Arakaki M., Garcia-Jacas N., Susanna A., Gitzendanner M.A., Soltis D.E., Soltis P. (2010). Isolation and characterization of novel microsatellite markers for *Arctium minus* (Compositae). Am. J. Bot..

[188] Miglani A., Manchanda R.K. (2014). Observational study of *Arctium lappa* in the treatment of acne vulgaris. Homeopathy.

[189] De Almeida A.B., Sanchez-Hidalgo M., Martin A.R., Luiz-Ferreira A., Trigo J.R., Vilegas W., dos Santos L.C., Souza-Brito A.R., de la Lastra C.A. (2013). Anti-inflammatory intestinal activity of *Arctium lappa* L. (Asteraceae) in TNBS colitis model. J. Ethnopharmacoly.

[190] Ahangarpour A., Heidari H., Oroojan A.A., Mirzavandi F., Nasr K.E., Mohammadi Z.D. (2017). Antidiabetic, hypolipidemic and hepatoprotective effects of *Arctium lappa* root’s hydro-alcoholic extract on nicotinamide-streptozotocin induced type 2 model of diabetes in male mice. Avicenna J. Phytomed..

[191] European Medicines Agency (2011). Community Herbal Monograph on Actium lappa L..

[192] Wang D., Bădărau A.S., Swamy M.K., Shaw S., Maggi F., da Silva L.E., López V., Yeung A., Mocan A., Atanasov A.G. (2019). Arctium species secondary metabolites chemodiversity and bioactivities. Front. Plant Sci..

[193] Su S., Cheng X., Wink M. (2015). Natural lignans from *Arctium lappa* modulate P-glycoprotein efflux function in multidrug resistant cancer cells. Phytomedicine.

[194] Lou Z., Li C., Kou X., Yu F., Wang H., Smith G.M., Zhu S. (2016). Antibacterial, antibiofilm effect of burdock (*Arctium lappa* L.) leaf fraction and its efficiency in meat preservation. J. Food Protect..

[195] Tang Y., Lou Z.X., Rahman M.R.T., Al-Hajj N.Q., Wang H. (2014). Chemical composition and anti-biofilm activity of burdock (*Arctium lappa* L. Asteraceae) leaf fractions against *Staphylococcus aureus*. Trop. J. Pharm. Res..

[196] Liu S.M., Chen K.S., Schliemann W., Strack D. (2005). Isolation and identification of arctiin and arctigenin in leaves of burdock (*Arctium lappla* L.) by polyamide column chromatography in combination with HPLC-ESI/MS. Phytochem. Anal..

[197] Lou C., Zhu Z., Zhao Y., Zhu R., Zhao H. (2017). Arctigenin, a lignan from *Arctium lappa* L., inhibits metastasis of human breast cancer cells through the downregulation of MMP-2/-9 and heparanase in MDA-MB-231 cells. Oncol. Rep..

[198] Susanti S., Iwasaki H., Inafuku M., Taira N., Oku H. (2013). Mechanism of arctigenin-mediated specific cytotoxicity against human lung adenocarcinoma cell lines. Phytomedicine.

[199] Huang K., Li L.A., Meng Y.G., You Y.Q., Fu X.Y., Song L. (2014). Arctigenin promotes apoptosis in ovarian cancer cells via the iNOS/NO/STAT3/survivin signalling. Basic Clin. Pharmacol. Toxicol..

[200] Predes F.S., Ruiz A.L.T.G., Carvalho J.E., Foglio M.A., Dolder H. (2011). Antioxidative and in vitro antiproliferative activity of *Arctium lappa* root extracts. BMC Compl. Altern. Med..

[201] Feng T., Cao W., Shen W., Zhang L., Gu X., Guo Y., Tsai H.I., Liu X., Li J., Zhang J. (2017). Arctigenin inhibits STAT3 and exhibits anticancer potential in human triple-negative breast cancer therapy. Oncotarget.

[202] Dribnenki J.C.P., McEachern S.F., Chen Y., Green A.G., Rashid K.Y. (2007). 2149 Solin (low linolenic flax). Can. J. Plant Sci..

[203] Goyal A., Sharma V., Upadhyay N., Gill S., Sihag M. (2014). Flax and flaxseed oil: An ancient medicine modern functional food. J. Food Sci. Technol..

[204] Tourre A., Xueming X. (2010). Flaxseed lignans: Source, biosynthesis, metabolism, antioxidant activity, bio-active components, and health benefits. Comp. Rev. Food Sci. Food Saf..

[205] Gebauer S.K., Psota T.L., Harris W.S., Kris-Etherton P.M. (2006). n-3 fatty acid dietary recommendations and food sources to achieve essentiality and cardiovascular benefits. Am. J. Clin. Nutr..

[206] Pellizzon M.A., Billheimer J.T., Bloedon L.T., Szapary P.O., Rader D.J. (2007). Flaxseed reduces plasma cholesterol levels in hypercholesterolemic mouse models. J. Am. Coll. Nutr..

[207] Simopoulos A.P. (2000). Human requirement for omega-3 polyunsaturated fatty acids. Poul. Sci..

[208] Gogus U., Smith C. (2010). n-3 Omega fatty acids: A review of current knowledge. Int. J. Food Sci. Technol..

[209] Chen J., Stavro P.M., Thompson L.U. (2002). Dietary flaxseed inhibits human breast cancer growth and metastasis and downregulates expression of insulin-like growth factor and epidermal growth factor receptor. Nutr. Cancer.

[210] Bergman J.M., Thompson L.U., Dabrosin C. (2007). Flaxseed and its lignans inhibit estradiol-induced growth, angiogenesis, and secretion of vascular endothelial growth factor in human breast cancer xenografts in vivo. Clin. Cancer Res..

[211] Truan J.S., Chen J.M., Thompson L.U. (2012). Comparative effects of sesame seed lignan and flaxseed lignan in reducing the growth of human breast tumors (MCF-7) at high levels of circulating estrogen in athymic mice. Nutr. Cancer.

[212] Puhalla S., Brufsky A., Davidson N. (2009). Adjuvant endocrine therapy for premenopausal women with breast cancer. Breast.

[213] VandeCreek L., Rogers E., Lester J. (1999). Use of alternative therapies among breast cancer outpatients compared with the general population. Altern. Ther. Health Med..

[214] Chen J., Power K., Mann J., Cheng A., Thompson L.U. (2007). Dietary flaxseed interaction with tamoxifen induced tumor regression in athymic mice with MCF-7 xenografts by downregulating the expression of estrogen related gene products and signal transduction pathways. Nutr. Cancer.

[215] Saggar J.K., Chen J., Corey P., Thompson L.U. (2009). Dietary flaxseed lignan or oil combined with tamoxifen treatment affects MCF-7 tumor growth through estrogen receptor- and growth factor-signaling pathways. Mol. Nutr. Food Res..

[216] Cotterchio M., Boucher B.A., Kreiger N., Mills C.A., Thompson L.U. (2008). Dietary phytoestrogen intake—Lignans and isoflavones—And breast cancer risk (Canada). Cancer Causes Control.

[217] Flower G., Fritz H., Balneaves L.G., Verma S., Skidmore B., Fernandes R., Kennedy D., Cooley K., Wong R., Sagar S. (2013). Flax and breast cancer: A systematic review. Integr. Cancer Ther..

[218] Buck K., Zaineddin A.K., Vrieling A., Linseisen J., Chang-Claude J. (2010). Meta-analyses of lignans and enterolignans in relation to breast cancer risk. Am. J. Clin. Nutr..

[219] Velentzis L.S., Cantwell M.M., Cardwell C., Keshtgar M.R., Leathem A.J., Woodside J.V. (2009). Lignans and breast cancer risk in pre- and post-menopausal women: Meta-analyses of observational studies. Br. J. Cancer.

[220] Khankari N.K., Bradshaw P.T., Steck S.E., He K., Olshan A.F., Shen J. (2015). Polyunsaturated fatty acid interactions and breast cancer incidence: A population-based case-control study on Long Island, New York. Ann. Epidemiol..

[221] Thanos J., Cotterchio M., Boucher B.A., Kreiger N., Thompson L.U. (2006). Adolescent dietary phytoestrogen intake and breast cancer risk (Canada). Cancer Causes Control.

[222] Touillaud M.S., Thiébaut A.C.M., Fournier A., Niravong M., Boutron-Ruault M.C., Clavel-Chapelon F. (2007). Dietary lignan intake and postmenopausal breast cancer risk by estrogen and progesterone receptor status. J. Natl. Cancer Inst..

[223] Buck K., Vrieling A., Zaineddin A.K., Becker S., Hüsing A., Kaaks R., Linseisen J., Flesch-Janys D., Chang-Claude J. (2011). Serum enterolactone and prognosis of postmenopausal breast cancer. J. Clin. Oncol..

[224] Zeleniuch-Jacquotte A., Adlercreutz H., Shore R.E., Koenig K.L., Kato I., Arslan A.A., Toniolo P. (2004). Circulating enterolactone and risk of breast cancer: A prospective study in New York. Br. J. Cancer.

[225] Yarnell E., Abascal K. (2011). *Nigella sativa*: Holy herb of the Middle East. Altern. Complement. Ther..

[226] Al-Jassir M.S. (1992). Chemical composition and microflora of black cumin (*Nigella sativa* L.) seeds growing in Saudi Arabia. Food Chem..

[227] Nickavar B., Mojab F., Javidnia K., Amoli M.A. (2003). Chemical composition of the fixed and volatile oils of *Nigella sativa* L. from Iran. Z. Naturforschung C.

[228] Bettaieb I., Bourgou S., Sriti J., Msaada K., Limam F., Marzouk B. (2011). Essential oils and fatty acids composition of Tunisian and Indian cumin (*Cuminum cyminum* L.) seeds: A comparative study. J. Sci. Food Agric..

[229] Ali B.H., Blunden G. (2003). Pharmacological and toxicological properties of *Nigella sativa*. Phytother. Res..

[230] Boskabady M.H., Shirmohammadi B. (2002). Effect of *Nigella sativa* on isolated guinea pig trachea. Arch. Iran. Med..

[231] Tavakkoli A., Ahmadi A., Razavi B.M., Hosseinzadeh H. (2017). Black seed (*Nigella sativa*) and its constituent thymoquinone as an antidote or a protective agent against natural or chemical toxicities. Iran. J. Pharm. Res..

[232] Badary O.A., Taha R.A., Gamal el-Din A.M., Abdel-Wahab M.H. (2003). Thymoquinone is a potent superoxide anion scavenger. Drug Chem. Toxicol..

[233] Wong R.S. (2011). Apoptosis in cancer: From pathogenesis to treatment. J. Exp. Clin. Cancer Res..

[234] Sundaravadivelu S., Raj S.K., Kumar B.S., Arumugamand P., Ragunathan P.P. (2019). Reverse screening bioinformatics approach to identify potential anti breast cancer targets using thymoquinone from neutraceuticals black Cumin il. Anticancer Agents Med. Chem..

[235] Ahmad A., Mishra R.K., Vyawahare A., Kumar A., Rehman M.U., Qamar W., Khan A.Q., Khan R. (2019). Thymoquinone (2-Isoprpyl-5-methyl-1, 4-benzoquinone) as a chemo-preventive/anticancer agent: Chemistry and biological effects. Saudi Pharm. J..

[236] Motaghed M., Al-Hassan F.M., Hamid S.S. (2013). Cellular responses with thymoquinone treatment in human breast cancer cell line MCF-7. Pharmacogn. Res..

[237] Dilshad A., Abulkhair O., Nemenqani D., Tamimi W. (2012). Antiproliferative properties of methanolic extract of *Nigella sativa* against the MDA-MB-231 cancer cell line. Asian Pac. J. Cancer Prev..

[238] Alhazmi M.I., Hasan T.N., Shafi G., Al-Assaf A.H., Alfawaz M.A., Alshatwi A.A. (2014). Roles of p53 and caspases in induction of apoptosis in MCF-7 breast cancer cells treated with a methanolic extract of *Nigella sativa* seeds. Asian Pac. J. Cancer Prev..

[239] Woo C.C., Loo S.Y., Gee V., Yap C.W., Sethi G., Kumar A.P., Tan K.H.B. (2011). Anticancer activity of thymoquinone in breast cancer cells: Possible involvement of PPAR-γ pathway. Biochem. Pharmacol..

[240] Jin X., Mu P. (2015). Targeting breast cancer metastasis. Breast Cancer Basic Clin. Res..

[241] Korak T., Ergül E., Sazci A. (2020). *Nigella sativa* and cancer: A review focusing on breast cancer, inhibition of metastasis and enhancement of natural killer cell cytotoxicity. Curr. Pharm. Biotechnol..

[242] Imran M., Rauf A., Khan I.A., Khan I.A., Shahbaz M., Qaisrani T.B., Fatmawati S., Abu-Izneid T., Imran A., Rahman K.U. (2018). Thymoquinone: A novel strategy to combat cancer: A review. Biomed. Pharmacother..

[243] Ait Mbarek L., Ait Mouse H., Elabbadi N., Bensalah M., Gamouh A., Aboufatima R., Benharre A., Chait A., Kamal M., Dalal A. (2007). Anti-tumor properties of blackseed (*Nigella sativa* L.) extracts. Braz. J. Med. Biol. Res..

[244] Khan M.A., Tania M., Wei C., Mei Z., Fu S., Cheng J., Xu J., Fu J. (2015). Thymoquinone inhibits cancer metastasis by downregulating TWIST1 expression to reduce epithelial to mesenchymal transition. Oncotarget.

[245] Baharetha H.M., Nassar Z.D., Aisha A.F., Ahamed M.B., Al-Suede F.S., Abd Kadir M.O., Ismail Z., Majid A.M. (2013). Proapoptotic and antimetastatic properties of supercritical CO2 extract of *Nigella sativa* Linn. against breast cancer cells. J. Med. Food.

[246] Lee S.R., Mun J.Y., Jeong M.S., Lee H.H., Roh Y.G., Kim W.T., Kim M.H., Heo J., Choi Y.H., Kim S.J. (2019). Thymoquinone-induced tristetraprolin inhibits tumor growth and metastasis through destabilization of MUC4 mRNA. Int. J. Mol. Sci..

[247] Shanmugam M.K., Arfuso F., Kumar A.P., Wang L., Goh B.C., Ahn K.S., Bishayee A., Sethi G. (2018). Modulation of diverse oncogenic transcription factors by thymoquinone, an essential oil compound isolated from the seeds of *Nigella sativa* Linn. Pharmacol. Res..

[248] Mollazadeh H., Afshari A.R., Hosseinzadeh H. (2017). Review on the potential therapeutic roles of *Nigella sativa* in the treatment of patients with cancer: Involvement of Apoptosis: Black cumin and cancer. J. Pharmacopunct..

[249] Linjawi S.A., Khalil W.K., Hassanane M.M., Ahmed E.S. (2015). Evaluation of the protective effect of *Nigella sativa* extract and its primary active component thymoquinone against DMBA-induced breast cancer in female rats. Arch. Med. Sci..

[250] Kabil N., Bayraktar R., Kahraman N., Mokhlis H.A., Calin G.A., Lopez-Berestein G., Ozpolat B. (2018). Thymoquinone inhibits cell proliferation, migration, and invasion by regulating the elongation factor 2 kinase (eEF-2K) signaling axis in triple-negative breast cancer. Breast Cancer Res. Treat..

[251] Woo C.C., Hsu A., Kumar A.P., Sethi G., Tan K.H. (2013). Thymoquinone inhibits tumor growth and induces apoptosis in a breast cancer xenograft mouse model: The role of p38 MAPK and ROS. PLoS ONE.

[252] Ong Y.S., Yazan L.S., Ng W.K., Noordin M.M., Sapuan S., Foo J.B., Tor Y.S. (2016). Acute and subacute toxicity profiles of thymoquinone-loaded nanostructured lipid carrier in BALB/c mice. Int. J. Nanomed..

[253] Suddek G.M. (2014). Protective role of thymoquinone against liver damage induced by tamoxifen in female rats. Can. J. Physiol. Pharmacol..

[254] Goyal S.N., Prajapati C.P., Gore P.R., Patil C.R., Mahajan U.B., Sharma C., Talla S.P., Ojha S.K. (2017). Therapeutic potential and pharmaceutical development of thymoqui none: A multi-targeted molecule of natural origin. Front. Pharmacol..

[255] Pathan S.A., Jain G.K., Zaidi S.M.A., Akhter S., Vohora D., Chander P., Kole P.L., Ahmad F.J., Khar R.K. (2011). Stability-indicating ultra-performance liquid chromatography method for the estimation of thymoquinone and its application in biopharmaceutical studies. Biomed. Chromatogr..

[256] Alkharfy K.M., Ahmad A., Khan R.M., Al-Shagha W.M. (2015). Pharmacokinetic plasma behaviors of intravenous and oral bioavailability of thymoquinone in a rabbit model. Eur. J. Drug Metab. Pharmacokinet..

[257] Poonia N., Kharb R., Lather V., Pandita D. (2016). Nanostructured lipid carriers: Versatile oral delivery vehicle. Future Sci. OA.

[258] Silva A.C., Santos D., Ferreira D., Lopes C.M. (2012). Lipid-based nano-carriers as an alternative for oral delivery of poorly water-soluble drugs: Peroral and mucosal routes. Curr. Med. Chem..

[259] Bhattacharya S., Ahir M., Patra P., Mukherjee S., Ghosh S., Mazumdar M., Chattopadhyay S., Das T., Chattopadhyay D., Adhikary A. (2015). PEGylated-thymoquinone-nanoparticle mediated retardation of breast cancer cell migration by deregulation of cytoskeletal actin polymerization through miR-34a. Biomaterials.

[260] Ng W.K., Yazan L.S., Yap L.H., Wan Nor Hafiza W.A., How C.W., Abdullah R. (2015). Thymoquinone-loaded nanostructured lipid carrier exhibited cytotoxicity towards breast cancer cell lines (MDA-MB-231 and MCF-7) and cervical cancer cell lines (HeLa and SiHa). Biomed Res. Int..

[261] Ong Y.S., Yazan L.S., Ng W.K., Abdullah R., Mustapha N.M., Sapuan S., Foo J.B., Tor Y.S., How C.W., Abd Rahman N. (2019). Thymoquinone loaded in nanostructured lipid carrier showed enhanced anticancer activity in 4T1 tumor-bearing mice. Nanomedicine.

[262] Dehghani H., Hashemi M., Entezari M., Mohsenifar A. (2015). The comparison of anticancer activity of thymoquinone and nanothymoquinone on human breast adenocarcinoma. Iran. J. Pharm. Res..

[263] Periasamy V.S., Athinarayanan J., Alshatwi A.A. (2016). Anticancer activity of an ultrasonic nanoemulsion formulation of *Nigella sativa* L. essential oil on human breast cancer cells. Ultrason. Sonochem..

[264] Aygun A., Gülbagca F., Ozer L.Y., Ustaoglu B., Altunoglu Y.C., Baloglu M.C., Atalar M.N., Alma M.H., Sen F. (2020). Biogenic platinum nanoparticles using black cumin seed and their potential usage as antimicrobial and anticancer agent. J. Pharm. Biomed. Anal..

[265] Rohini B., Akther T., Waseem M., Khan J., Kashif M., Hemalatha S. (2019). AgNPs from *Nigella sativa* control breast cancer: An in vitro study. J. Environ. Pathol. Toxicol. Oncol..

[266] Effenberger-Neidnicht K., Schobert R. (2011). Combinatorial effects of thymoquinone on the anti-cancer activity of doxorubicin. Cancer Chemother. Pharmacol..

[267] Mahmoud S.S., Torchilin V.P. (2013). Hormetic/cytotoxic effects of *Nigella sativa* seed alcoholic and aqueous extracts on MCF-7 breast cancer cells alone or in combination with doxorubicin. Cell Biochem. Biophys..

[268] Harper N.W., Hodges K.B., Stewart R.L., Wu J., Huang B., O’Connor K.L., Romond E.H. (2019). Adjuvant treatment of triple-negative metaplastic breast cancer with weekly paclitaxel and platinum chemotherapy: Retrospective case review from a single institution. Clin. Breast Cancer.

[269] Şakalar Ç., İzgi K., İskender B., Sezen S., Aksu H., Çakır M., Kurt B., Turan A., Canatan H. (2016). The combination of thymoquinone and paclitaxel shows anti-tumor activity through the interplay with apoptosis network in triple-negative breast cancer. Tumour Biol..

[270] Perroud H.A., Alasino C.M., Rico M.J., Mainetti L.E., Queralt F., Pezzotto S.M., Rozados V.R., Scharovsky O.G. (2016). Metastatic breast cancer patients treated with low-dose metronomic chemotherapy with cyclophosphamide and celecoxib: Clinical outcomes and biomarkers of response. Cancer Chemother. Pharmacol..

[271] Al-Mutairi A., Rahman A., Rao M.S. (2020). Low doses of thymoquinone and ferulic acid in combination effectively inhibit proliferation of cultured MDA-MB 231 breast adenocarcinoma cells. Nutr. Cancer.

[272] Talib W.H. (2017). Regressions of breast carcinoma syngraft following treatment with piperine in combination with thymoquinone. Sci. Pharm..

[273] Al-Amri A.A., Bamoasa A.O. (2009). Phase I safety and clinical activity of thymoquinone in patients with advanced refractory malignant disease. Shiraz E Med. J..

[274] Odeh F., Ismail S.I., Abu-Dahab R., Mahmoud I.S., Al Bawab A. (2012). Thymoquinone in liposomes: A study of loading efficiency and biological activity towards breast cancer. Drug Deliv..

